# Identification of New Shikonin Derivatives as Antitumor Agents Targeting STAT3 SH2 Domain

**DOI:** 10.1038/s41598-017-02671-7

**Published:** 2017-06-06

**Authors:** Han-Yue Qiu, Xiang Zhu, Yue-Lin Luo, Hong-Yan Lin, Cheng-Yi Tang, Jin-Liang Qi, Yan-Jun Pang, Rong-Wu Yang, Gui-Hua Lu, Xiao-Ming Wang, Yong-Hua Yang

**Affiliations:** 1 State Key Laboratory of Pharmaceutical Biotechnology, NJU-NJFU Joint Institute of Plant Molecular Biology, Nanjing University, Nanjing, 210023 China; 2 Co-Innovation Center for Sustainable Forestry in Southern China, Nanjing Forestry University, Nanjing, 210037 China

## Abstract

Signal transducer and activator of transcription 3 (STAT3) is hyper-activated in diversiform human tumors and has been validated as an attractive therapeutic target. Current research showed that a natural product, shikonin, along with its synthetic analogues, is able to inhibit the activity of STAT3 potently. The potential space of shikonin in developing novel anti-cancer agents encouraged us to carry out the investigation of the probable binding mode with STAT3. From this foundation, we have designed new types of STAT3 SH2 inhibitors. Combined simulations were performed to filter for the lead compound, which was then substituted, synthesized and evaluated by a variety of bioassays. Among the entities, PMM-172 exhibited the best anti-proliferative activity against MDA-MB-231 cells with IC_50_ value 1.98 ± 0.49 *μ*M. Besides, it was identified to decrease luciferase activity, induce cell apoptosis and reduce mitochondrial transmembrane potential in MDA-MB-231 cells. Also, PMM-172 inhibited constitutive/inducible STAT3 activation without affecting STAT1 and STAT5 in MDA-MB-231 cells, and had no effect in non-tumorigenic MCF-10A cells. Moreover, PMM-172 suppressed STAT3 nuclear localization and STAT3 downstream target genes expression. Overall, these results indicate that the antitumor activity of PMM-172 is at least partially due to inhibition of STAT3 in breast cancer cells.

## Introduction

The STAT family of proteins is a key mediator transferring extracellular signals into cell nucleus to regulate various cellular processes including proliferation, apoptosis, angiogenesis and other biological activities^1, 2^. This family consists of seven members which share similarity in structure, albeit they are encoded by disparate genes^3^. One of the most important family members, the STAT3 protein, is phosphorylated and activated by its upstream kinases such as FAK, JAK and Src^4^. The phosphorylation of STAT3 leads to the dimerization of its monomers through SH2 domains and after then the STAT3 dimers translocate into cell nucleus to perform their biological functions as gene expression regulator^5, 6^.

Compelling research shows that aberrantly activated STAT3 is tightly connected with cell dysfunction which causes morbid processes such as tumorigenesis and malignant transformation^7^. Clinically, the overexpression or constitutively activation of this transcription factor is observed in 70% of human solid and hematological tumors. In turn, inhibiting the activity of STAT3 has proved its potency to hinder the tumor process, both *in vitro* and *in vivo* ^8, 9^. Thus STAT3 is considered a promising molecular target in cancer therapies and attracts considerable interest of medicinal chemists^10, 11^.

Current drug design strategy for suppressing STAT3 activity is mainly comprised of two methods. One is to inhibit its upstream kinases so that the phosphorylation process of STAT3 is thwarted. However, since these kinases target multiple downstream proteins, undesirable adverse effects might appear when other pathways are down-regulated. The other one is to directly suppress the activity of STAT3^12–14^. Compared with the former method, this strategy is more appealing as normal cell functions are minimally disturbed^15^. To date, a number of STAT3 inhibitors have been identified and being tested in different stages^3, 16, 17^; still and all, inevitable attrition is predictable during the developing process, and the situation is worse off taking the tumorous inherent tendency of getting resistant and relapsing into consideration. The need of a larger candidate pool applies to almost every therapy strategy of tumor, which is also true for targeting STAT3.

**Figure 1:**
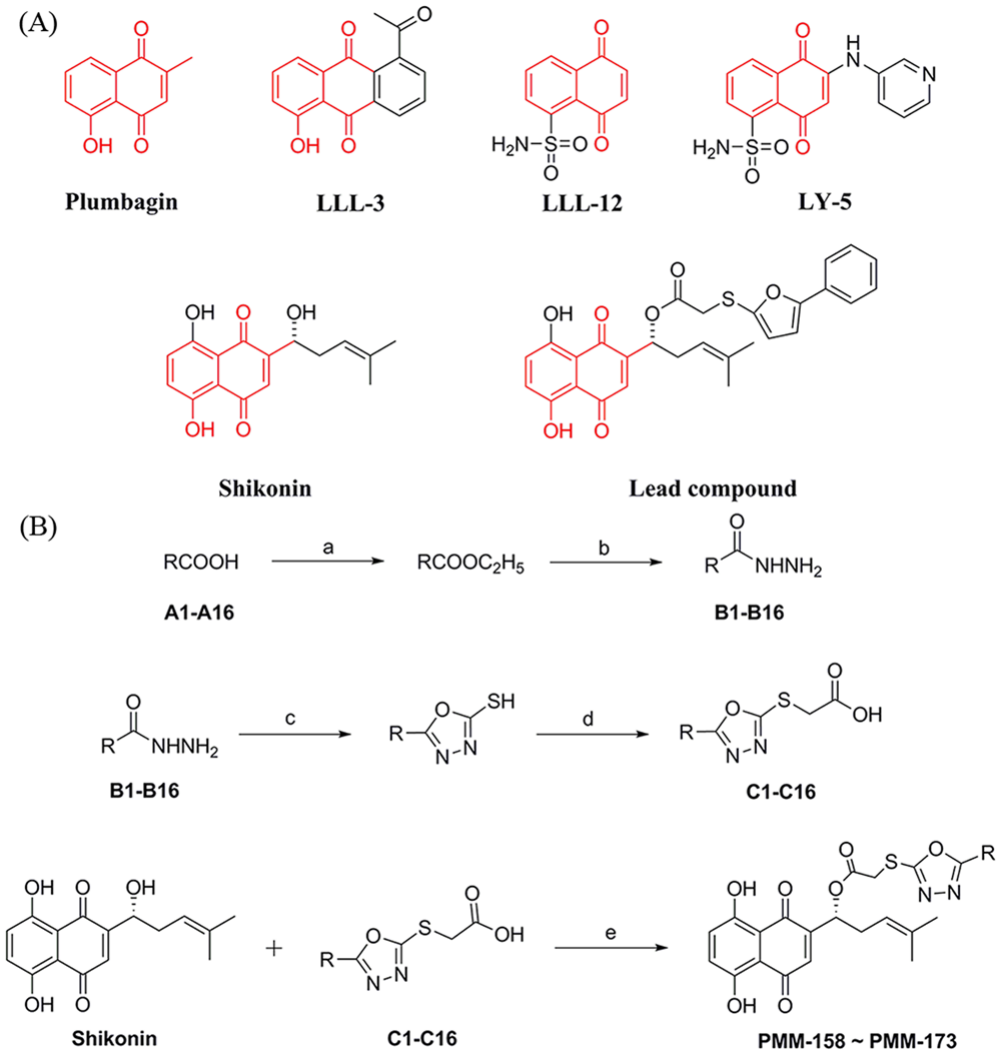
(**A**) Reported synthetic STAT3 inhibitors and SHK with lead compound. The analogous structure for these compounds is colored in red. (**B**) Synthesis of compounds PMM-158~PMM-173. Reagents and conditions: (a) concentrated sulfuric acid, ethanol, reflux, 24 h; (b) NH_2_NH_2_-H_2_O (85%), ethanol, reflux, 12 h; (c) (1) CS_2_/KOH, ethanol (95%), reflux, 24 h; (2) HCl, PH 5–6; (d) ClCH_2_COOH, ethanol, reflux, 12 h; (e) DCC, DMAP, CH_2_Cl_2_, 0 °C, 8 h.

Recent research showed that shikonin (SHK), the main efficacy component of the Chinese herbal medicine *Lithospermum erythrorhizon*, is capable to block the STAT3 pathway^18–20^. Especially in breast cancer models, SHK exhibited impressive pharmacological characters which are related with its effect on STAT3^21^. Besides, recent reports have revealed that many synthetic STAT3 inhibitors, such as plumbagin and LLL-12 (Fig. 1A)^22–25^, share the same backbone with SHK, hinting a scaffold bias for the target STAT3. Hence SHK might be a good starting point for modification to achieve new agents with improved profile against STAT3 and relating tumors^26–29^.

In this work we primarily explored the potential binding mode of SHK with STAT3. On the basis we employed Scaffold Growth strategy to furnish a library of modifications, which were then selected by virtual screen^30^. Molecular dynamics simulation was also performed to validate the docking results. The outcome showed that the optimization provided several entities with improved potency compared with their scaffold SHK.

## Results

### Chemistry

The synthesis of compounds PMM-158~PMM-173 followed the general pathway outlined in Fig. 1B. All synthesized compounds were reported and characterized for the first time, and gave satisfactory analytical and spectroscopic data. ^1^H NMR and ESI-MS spectra were in full accordance with the assigned structures (shown in the Supplementary Information).

### Molecular docking and scaffold modification

**Figure 2:**
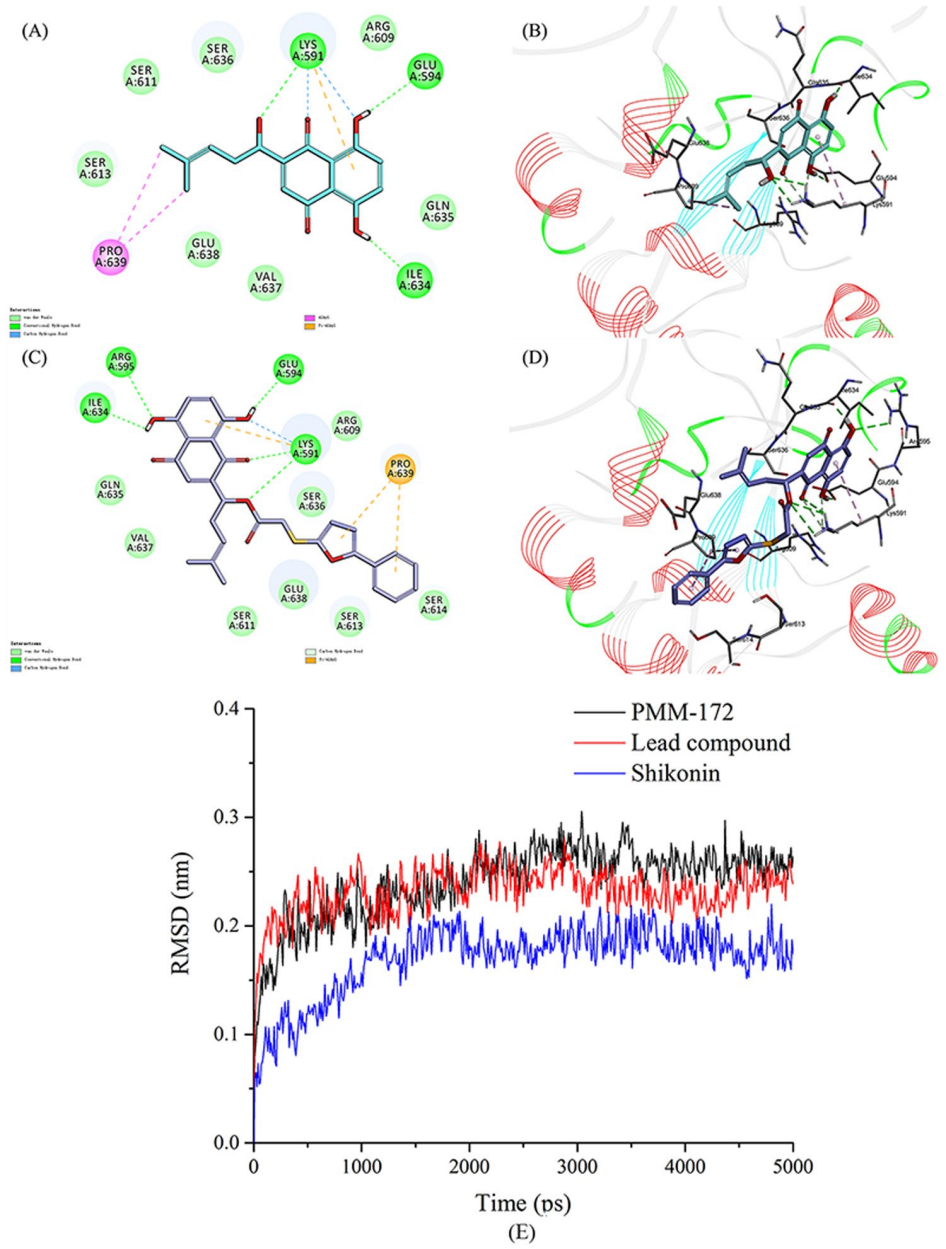
Binding mode of SHK and lead compound PMM-158 with STAT3 (PDB code: 1BG1). (**A**) 2D diagram of the interaction between SHK and amino acid residues of the nearby active site. (**B**) 3D diagram of SHK inserted in the STAT3 binding site: for clarity, 3D structure of the protein is presented in line ribbon and only interacting residues are displayed. (**C**) 2D diagram of the interaction between PMM-158 and amino acid residues of the nearby active site. (**D**) 3D diagram of PMM-158 inserted in the STAT3 binding site: for clarity, 3D structure of the protein is presented in line ribbon and only interacting residues are displayed. (**E**)The RMSDs of the three studied complexes obtained during 5 ns of MD simulations, SHK (blue), lead compound (red) and PMM-172 (black).

The homodimerization of STAT3 is mediated by the PPI (protein-protein interaction) between SH2 domains of each STAT3 monomers, and the pTyr705 plays a vital role in this process. In the SH2 domains of STAT3, three adjacent binding subpockets were explored as the binding hot spots, namely pY-X (Δ 592–605), pY + 0 (591, Δ 609–620), and pY + 1 (Δ 626–639) site. The site pY + 0 binds with pTyr705 and contains polar residues responsible for hydrogen bonding and electrostatic interactions, and the other two subpockets pY + 1 and pY-X are formed by hydrophobic residues. Most of the known STAT3 inhibitors targeting the SH2 domain bind at least two of these three subpockets. In this work, docking simulations were performed iteratively. We primarily explored the binding mode of SHK with STAT3, as shown in Fig. 2A and Fig. 2B, the starting SHK mainly located in the pY-X and pY + 0 pockets hydrogen bonding with LYS 591, GLU 594 and ILE 634. This predicted mode is consistent with those gained by the mentioned SHK analogues, hinting a consensus scaffold bias in this pocket area. After then, modification was made on SHK backbone to establish a library of small molecules in order to screen for potential compounds. All the outcomes had better binding affinities than the scaffold SHK. Docking screening was employed to rank these candidates. In addition to the virtual screening, the synthetic accessibility for these candidates was taken into consideration. At last, the lead compound illustrated in Fig. 1A was validated. It was then substituted to furnish a class of derivatives which were also tested by docking simulation. The docking results are summarized in Table 1, the lead compound occupied the same two subpockets pY-X and pY + 0 assembling its scaffold SHK. Besides, it interacts with the same residues LYS 591, GLU 594 and ILE 634 comparing with SHK (Fig. 2C,D), however one more hydrogen bonds with residue ARG595 was formed, along with the coming up of additional interactions induced by the modification part.

**Table 1:** The interaction energy and anti-proliferative activity against non-tumorigenic MCF-10A cells of the target compounds.

Compd	R	Interaction energy ΔG_b_ (kcal/mol)	CC_50_ (μM) MCF-10A
PMM-158		−56.6335	92.5 ± 0.27
PMM-159		−55.7343	89.5 ± 0.99
PMM-160		−57.7694	68.3 ± 0.27
PMM-161		−59.1773	84.5 ± 0.74
PMM-162		−59.4741	91.9 ± 1.21
PMM-163		−61.1946	86.2 ± 0.33
PMM-164		−59.9377	74.6 ± 0.55
PMM-165		−61.361	83.7 ± 1.28
PMM-166		−56.5488	69.9 ± 0.58
PMM-167		−59.8725	72.5 ± 1.53
PMM-168		−60.3577	80.6 ± 1.07
PMM-169		−60.9084	99.5 ± 0.49
PMM-170		−61.8761	94.6 ± 0.83
PMM-171		−62.6852	76.2 ± 1.15
**PMM-172**		−63.1153	95.4 ± 0.62
PMM-173		−59.983	88.9 ± 0.34
Shikonin		−59.4517	23.5 ± 1.01

### MD trajectory analysis

In this study, we analyzed the dynamic behavior in the ligand-binding site of the STAT3-inhibitor complexes. It’s well established that the root mean square deviation (RMSD) fluctuation is an important criterion to gauge whether the protein-ligand system was stable. During the process of MD simulation, the maximum RMSD for each system was limited to 0.3 nm (Fig. 2E). The RMSDs of all the systems gradually became stable after 1 ns, indicating that they reached equilibrium. It has been shown that PMM-172 became stable quickly and could present an equilibrium state for more than 3 ns. Notably, we found that the RMSD of PMM-172 remains stable throughout the whole process of simulation, suggesting its distinct comparative advantage against other compounds.

### PMM-172 inhibited cell growth and STAT3 transcriptional activity

**Figure 3:**
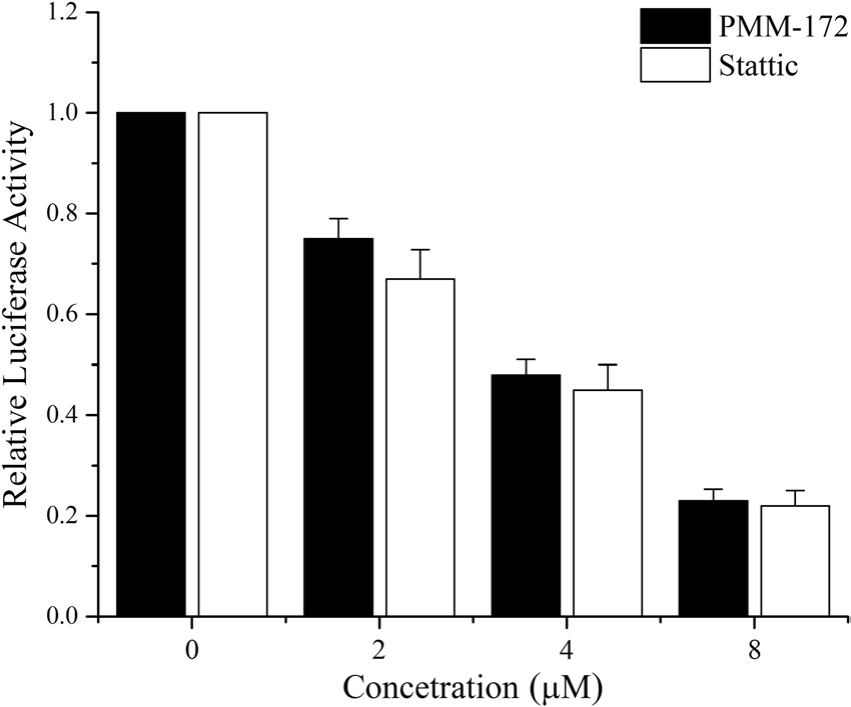
MDA-MB-231 cells were transfected with STAT3-TATA-Luc reporter and Renilla luciferase reporter plasmids. After 24 h, cells were treated with indicated concentrations of PMM-172 or Stattic for 24 h and then luciferase intensity was measured. Results are fold change ± SD of three independent experiments, *P < 0.05, **P < 0.01.

Initial experiments were performed for evaluation of growth inhibition by the new compounds against six human breast cancer cell lines, including MCF-7 (ER+/PR+, HER-2-), BT474 (ER+/PR+, HER-2+), SKBR-3 (ER-/PR-/HER-2+), MDA-MB-436 (ER-/PR-/HER-2-), MDA-MB-231 (ER-/PR-/HER-2-), MDA-MB-468 (ER-/PR-/HER-2-) and non-tumorigenic MCF-10A cells. After 24 h treatment by the obtained compounds, the inhibition was assessed and the IC_50_ was summarized. The results shown in Table 1 indicated that all compounds had lower toxicity against the non-tumorigenic MCF-10A cells than SHK itself. As shown in Table 2, the new synthetic compounds showed high sensitivity to three triple negative cells and HER2 over-expression cells than to the other two subtypes. Fortunately, we obtained some compounds with strong cytotoxicity toward human breast cancer cells while avoiding non-tumorigenic MCF-10A cells. Among them, PMM-172 (IC_50_ = 1.98 ± 0.49 *μ*M) showed better anti-proliferative activity against MDA-MB-231 cells than SHK (IC_50_ = 2.88 ± 0.25 *μ*M). Subsequently, the inhibition of STAT3 activation by PMM-172 and Stattic was evaluated using a dual-luciferase assay. The results (Fig. 3) suggested that PMM-172 decreased the STAT3 luciferase activity in a dose-dependent manner, while the effects of Stattic showed only a slight advantage over PMM-172.

**Table 2:** Anti-proliferative activity against six kinds of breast cancer cells by all compounds.

Compound	IC_50_ (*μ*M)					
	MCF-7	BT-474	SKBR-3	MDA-MB-436	MDA-MB-231	MDA-MB-468
PMM-158	12.1 ± 0.42	15.6 ± 1.03	11.5 ± 0.74	10.1 ± 0.92	9.17 ± 0.99	10.6 ± 0.65
PMM-159	9.22 ± 0.13	10.8 ± 0.94	8.95 ± 0.69	8.49 ± 0.78	7.24 ± 0.82	8.23 ± 1.27
PMM-160	15.3 ± 0.52	18.3 ± 1.24	10.5 ± 0.88	11.8 ± 0.86	9.66 ± 1.21	12.4 ± 0.52
PMM-161	9.81 ± 0.49	11.1 ± 1.12	9.29 ± 0.66	9.55 ± 0.83	8.58 ± 0.53	8.97 ± 1.05
PMM-162	9.01 ± 0.92	9.54 ± 0.91	8.03 ± 0.83	7.28 ± 0.77	6.14 ± 1.05	6.85 ± 0.92
PMM-163	8.69 ± 0.83	9.27 ± 0.86	7.56 ± 0.72	7.09 ± 0.63	6.04 ± 0.59	7.83 ± 0.55
PMM-164	5.71 ± 0.52	7.09 ± 0.65	4.49 ± 0.58	4.41 ± 0.55	3.76 ± 1.02	3.94 ± 0.73
PMM-165	9.38 ± 0.66	9.22 ± 0.98	6.23 ± 0.73	5.97 ± 0.67	5.47 ± 1.01	6.58 ± 0.15
PMM-166	6.41 ± 0.75	6.91 ± 0.73	4.87 ± 0.58	4.66 ± 0.59	3.64 ± 0.91	4.07 ± 0.66
PMM-167	15.3 ± 0.61	13.7 ± 0.92	9.76 ± 0.95	10.6 ± 0.82	9.01 ± 0.56	12.2 ± 0.42
PMM-168	9.27 ± 1.12	10.6 ± 0.81	8.16 ± 0.71	7.87 ± 0.91	6.09 ± 1.04	8.42 ± 0.86
PMM-169	8.12 ± 0.98	8.36 ± 0.97	6.95 ± 0.82	7.36 ± 0.87	5.33 ± 0.77	7.22 ± 1.11
PMM-170	9.73 ± 1.03	12.7 ± 1.06	8.27 ± 0.69	6.88 ± 0.73	4.31 ± 0.65	8.44 ± 0.78
PMM-171	8.71 ± 0.67	8.59 ± 0.78	5.89 ± 0.64	6.49 ± 0.68	3.14 ± 1.02	6.95 ± 0.46
**PMM-172**	4.87 ± 0.55	5.26 ± 0.66	3.14 ± 0.46	3.07 ± 0.47	1.98 ± 0.49	3.24 ± 0.27
PMM-173	5.58 ± 0.83	6.03 ± 0.71	3.59 ± 0.49	4.06 ± 0.53	2.59 ± 0.19	3.76 ± 0.88
Shikonin	4.57 ± 0.69	5.74 ± 0.66	3.75 ± 0.52	3.28 ± 0.41	2.88 ± 0.25	3.61 ± 0.34
Stattic	5.85 ± 0.72	5.11 ± 0.57	3.39 ± 0.56	3.76 ± 0.50	3.18 ± 0.33	3.54 ± 0.42

### PMM-172 induced apoptosis in MDA-MB-231 cells in dose- and time- dependent manners

**Figure 4:**
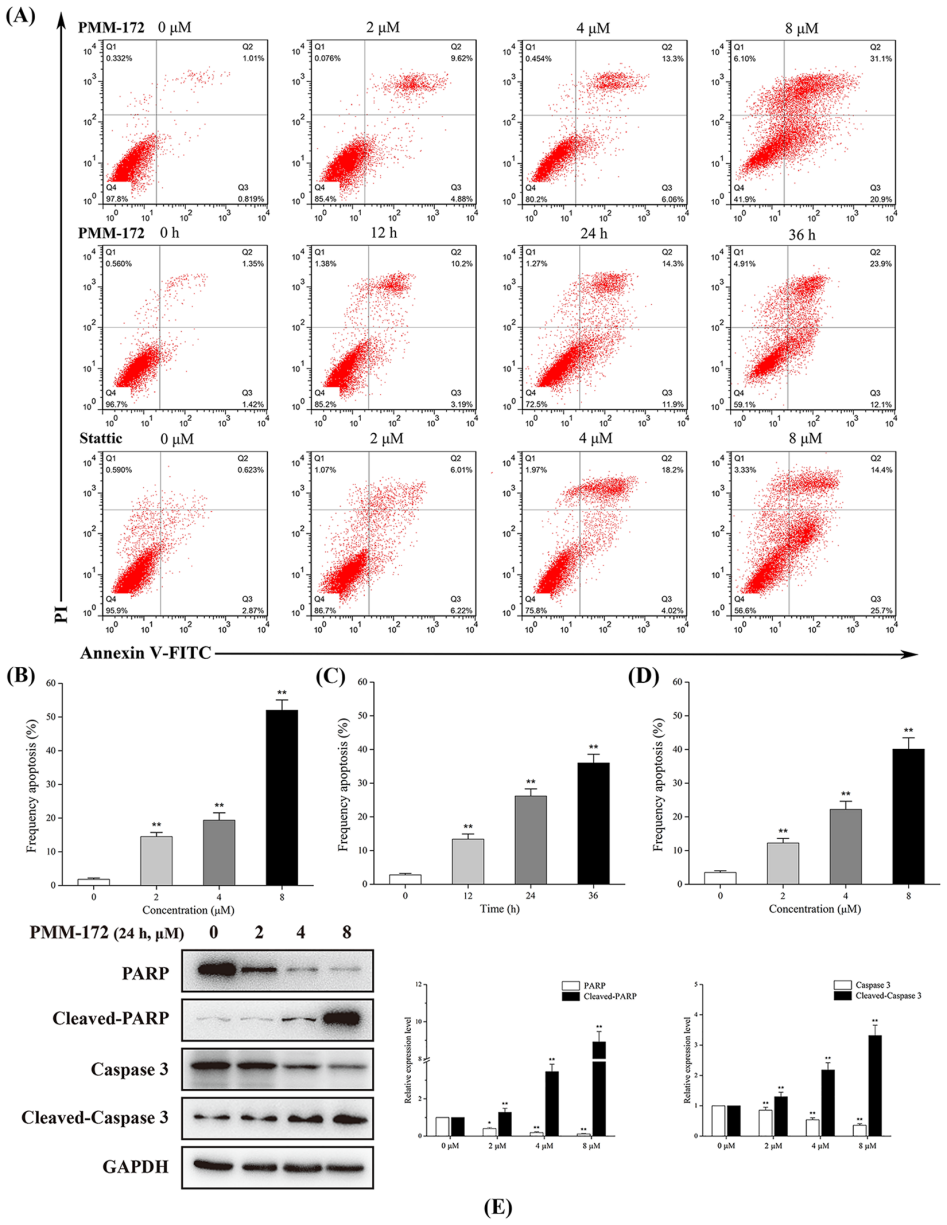
The effect of PMM-172 on the cell apoptosis of MDA-MB-231 cells. (**A**) MDA-MB-231 cells were treated with various concentrations of PMM-172 or Stattic for 24 h, or treated with 4 *μ*M PMM-172 for different time. Apoptosis was analyzed by flow cytometry by using Annexin V/PI double staining. (**B**) The percentage of apoptotic cells in MDA-MB-231 cells treated with various concentrations of PMM-172 for 24 h. (**C**) The percentage of apoptotic cells in MDA-MB-231 cells treated with 4 *μ*M PMM-172 for different time. (**D**) The percentage of apoptotic cells in MDA-MB-231 cells treated with various concentrations of Stattic for 24 h. (**E**) PARP and caspase 3 cleavage in MDA-MB-231 cells after treated with various concentrations of PMM-172 for 24 h were detected by using Western blot analysis. The relative expression level was analyzed by Image J software. Data were shown as mean ± SD of three independent experiments. *P < 0.05, **P < 0.01. Cropped blots/gels were used in the figure and the gels had been run under the same experimental conditions; the full-length blots/gels are presented in Supplementary Figure S1.

To determine whether PMM-172 induced apoptosis, Annexin V/PI double staining assay was conducted. MDA-MB-231 cells were treated with various concentrations of PMM-172 for 24 h, and the percentage of apoptotic cells was markedly elevated in a dose-dependent manner. Likewise, MDA-MB-231 cells were treated with corresponding doses of Stattic. As shown in Fig. 4A, increased apoptotic rate was detected after cells being treated with escalating concentrations of PMM-172. The percentages of cell apoptosis 3.12%, 8.12%, 16.81%, 62.74% corresponded to the concentration of PMM-172 0, 2, 4 and 8 *μ*M, respectively, including both the early and the late apoptotic cells. Clearly, PMM-172 can cause cell apoptosis more effectively than Stattic. Moreover, an extend time-course apoptosis analysis of MDA-MB-231 cells treated with 4 *μ*M PMM-172 was carry out. The results indicated that longer treatment periods showed an increasing tendency in the percentage of apoptotic cells. To further assess the pro-apoptotic effect of PMM-172, we evaluated the cleavage of poly (ADP-ribose) polymerase (PARP) and caspase 3 in MDA-MB-231 cells after 24 h treatment with PMM-172. In this case, the active form of PARP involved in DNA repair and programmed cell death is considered as the hallmark of apoptosis. The results of western blot showed that PMM-172 could induce PARP and caspase 3 cleavage in MDA-MB-231 cells in a dose-dependent, which were in accordance with the above results (Fig. 4E). Taken together, we obtained the conclusion that PMM-172 induced apparent apoptosis in MDA-MB-231 cells in dose- and time- dependent manners.

### PMM-172 reduced mitochondrial transmembrane potential in MDA-MB-231 cells

**Figure 5:**
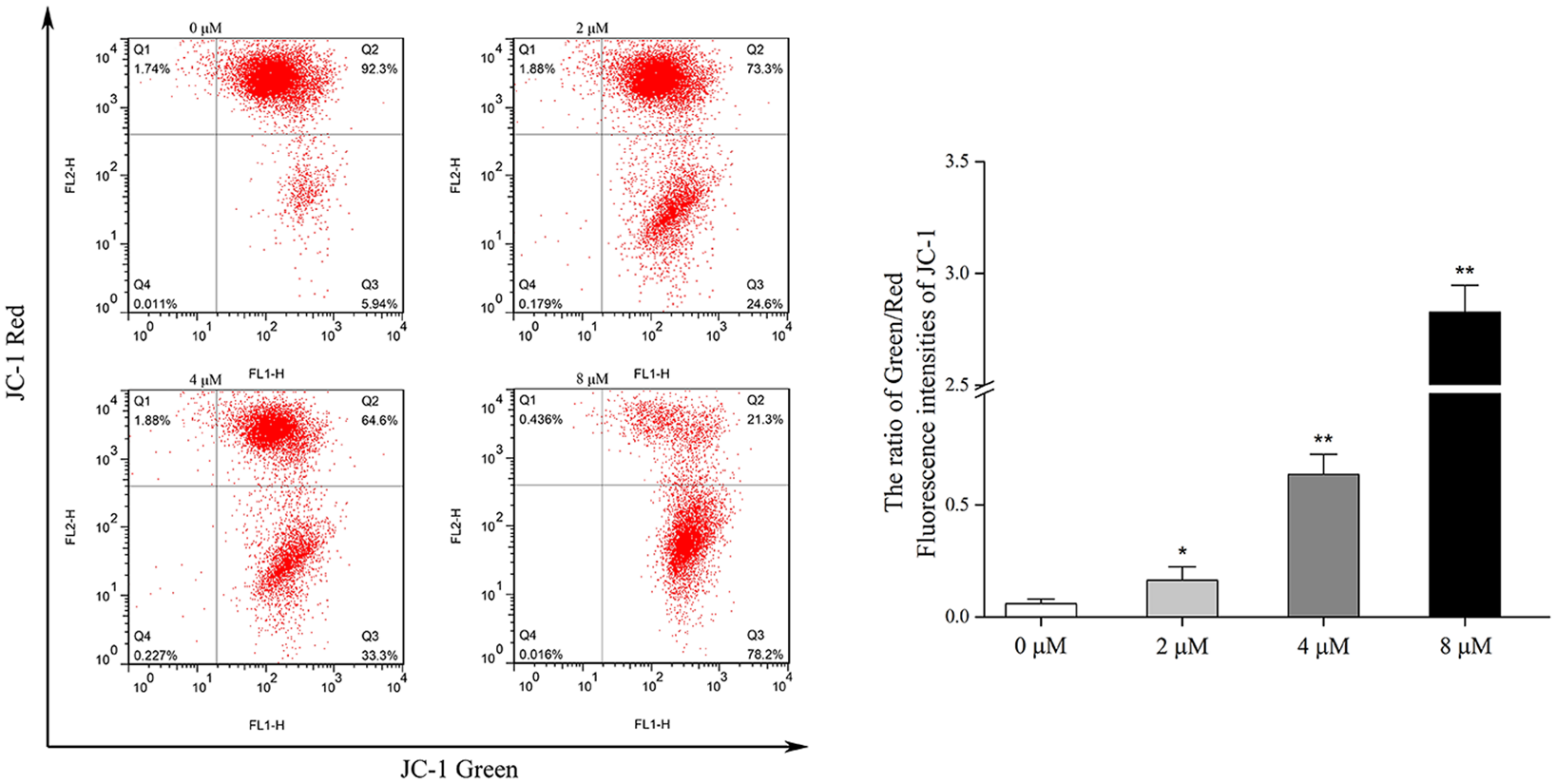
Mitochondrial transmembrane potential was analysed in PMM-172-treated MDA-MB-231 cells by JC-1 staining.

Subsequently, the mitochondrial transmembrane potential was monitored using JC-1 staining. The Fig. 5 shows representative JC-1 fluorescence in both FL-1 and FL-2 channels. Red fluorescence represents the mitochondrial aggregate form of JC-1 with high ∆*Ψ*m in normal cells. Green fluorescence represents the monomeric form of JC-1 with low ∆*Ψ*m in apoptotic cells. However, cells treated with PMM-172 exhibited increased green fluorescence and decreased red fluorescence, suggesting the depolarization of mitochondria and reduced ∆*Ψ*m. The ratio of cells with high membrane potential exposed to 4 *μ*M and 8 *μ*M PMM-172 decreased from 92.3% to 64.6% and 21.3% after 4 h treatment.

### PMM-172 reduced constitutive and inducible STAT3 activation in MDA-MB-231 cells

**Figure 6:**
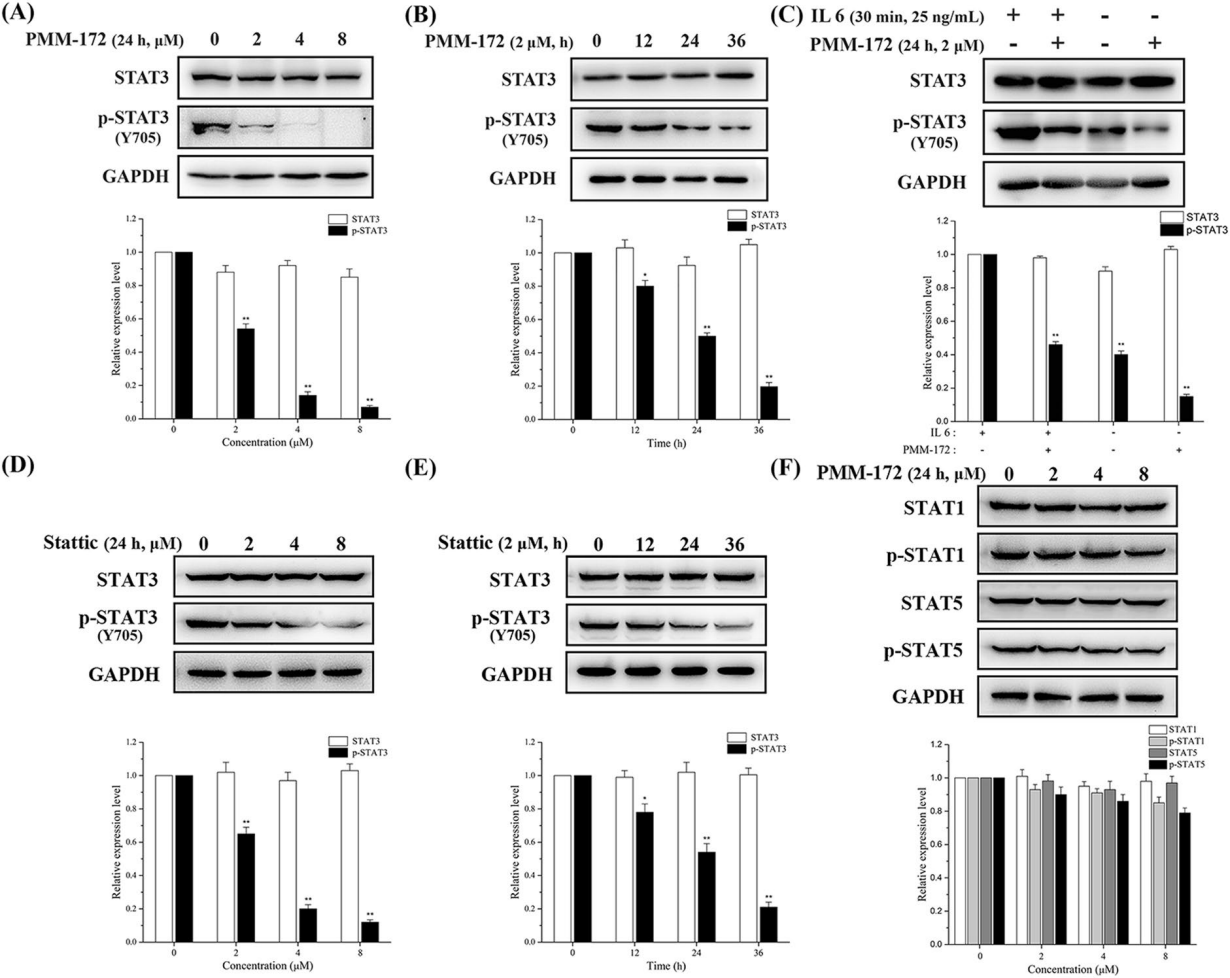
(**A**) PMM-172 suppressed constitutive pSTAT3 (Tyr705) in a dose-dependent manner. (**B**) PMM-172 suppressed constitutive pSTAT3 (Tyr705) in a time-dependent manner. (**C**) MDA-MB-231 cells were treated with IL-6 (20 ng/mL) or vehicle for 30 min, with or without pretreatment with PMM-172 (2 *μ*M) for 24 h. PMM-172 inhibited IL-6 induced phosphorylation of STAT3. (**D**) Stattic suppressed constitutive pSTAT3 (Tyr705) in a dose-dependent manner. (**E**) Stattic suppressed constitutive pSTAT3 (Tyr705) in a time-dependent manner. (**F**) Tyrosine phosphorylation of two STAT family proteins STAT1 and STAT5 were detected with specific antibodies. PMM-172 inhibited neither the phosphorylation nor expression of STAT1 and STAT5. Data were shown as mean ± SD of three independent experiments. *P < 0.05, **P < 0.01. Cropped blots/gels were used in the figure and the gels had been run under the same experimental conditions; the full-length blots/gels are presented in Supplementary Figure S2.

It has been well recognized that the aberrant activation of STAT3 contributes to malignant transformation and tumorgenesis, and its activity is ultimately dependent upon phosphorylation of residue Y705. Hence the Y705-phosphorylated level of STAT3 is an important metric to evaluate the inhibitory efficacy of STAT3 antagonists. In this study, the ability of PMM-172 and Stattic inhibiting the constitutive STAT3 activation in MDA-MB-231 cells was investigated. Western blot analysis (Fig. 6A and D) revealed that PMM-172 and Stattic could reduce the level of Y705-phosphorylated STAT3 in MDA-MB-231 cells in a dose-dependent manner, and had no effect on STAT3 expression. By comparison, we found that the inhibitory effect of PMM-172 was more potent than Stattic. We further measured STAT3 phosphorylation at Tyr705 in a time-course experiment. As shown in Fig. 6B and E, the phosphorylation of residue Tyr705 in STAT3 is reduced with the longer treatment of PMM-172 or Stattic.

We further investigated whether PMM-172 could inhibit IL-6-induced STAT3 phosphorylation in MDA-MB-231 cells. As shown in Fig. 6C, IL-6 stimulation on MDA-MB-231 cells increased the levels of phosphor-STAT3 while the pretreatment of PMM-172 suppressed IL-6-induced STAT3 phosphorylation without affecting STAT3 expression.

Subsequently, we evaluated the phosphorylation level of other STAT family proteins including STAT1 and STAT5. The results (Fig. 6F) showed PMM-172 inhibited neither the phosphorylation nor expression of STAT1 and STAT5, indicating that the inhibition of STAT3 activation by PMM-172 may be specific.

### PMM-172 suppressed STAT3 nuclear localization

**Figure 7:**
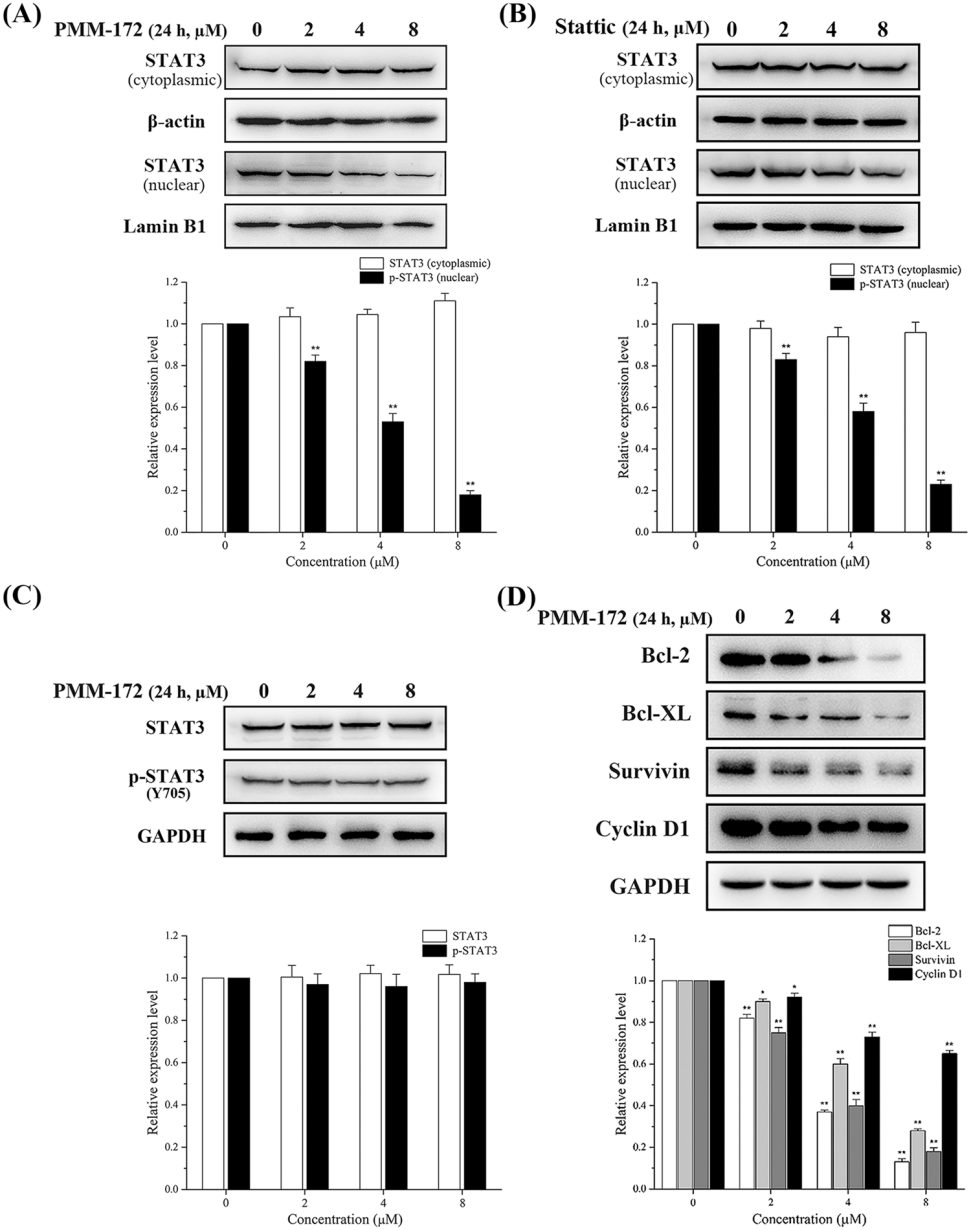
MDA-MB-231 cells were treated with indicated concentrations of PMM-172 or Stattic for 24 h, the expression levels of STAT3 in cytoplasmic and nuclear extracts were examined and relative expression levels were analyzed by Image J software. β-actin and Lamin B1 served as markers of cytoplasmic and nuclear extractions, respectively. (**A**) PMM-172 suppressed STAT3 nuclear localization. (**B**) Stattic suppressed STAT3 nuclear localization. (**C**) MCF-10A cells were treated with the indicated concentrations of PMM-172 for 24 h. PMM-172 had no effect on the level of the pSTAT3 level in MCF-10A cells. (**D**) PMM-172 down-regulated the expression of STAT3 target genes. Data were shown as mean ± SD from three independent experiments, **P < 0.01. Cropped blots/gels were used in the figure and the gels had been run under the same experimental conditions; the full-length blots/gels are presented in Supplementary Figure S3.

It is known that the dimerization of STAT3 is induced by the phosphorylation at residue Tyr 705, which then leads to its nuclear translocation. Herein, we investigated the effect of PMM-172 and Stattic on STAT3 nuclear translocation. The cytoplasmic and nuclear proteins of treated cells were extracted and the expressions of STAT3 in both fractions were examined. The results (Fig. 7A) showed that the level of STAT3 in the nuclear fraction was decreased by PMM-172 in a dose-dependent manner, while the level of STAT3 was slightly increased in the cytoplasmic fraction. As shown in Fig. 7B, the effect of PMM-172 to suppress STAT3 nuclear localization showed a slight advantage over Stattic.

### PMMB-172 does not suppress STAT3 activation nor viability in normal cells

In the following work, we examined whether PMM-172 could affect the activity of STAT3 and viability in non-cancer cells. The non-tumorigenic MCF-10A epithelial cells were employed to be treated with increasing doses of PMM-172 to conduct this assay. It is noteworthy that PMM-172 had no effect on the level of the pSTAT3 level in MCF-10A cells, and the expression levels of total STAT3 kept unchanged (Fig. 7C). The cell viability was evaluated using MTT assay, and the results revealed that the growth of MCF-10A cells was barely inhibited by PMM-172 (Table 1). Taken together, these results indicated that PMM-172 primarily suppressed STAT3 activation and viability in STAT3-depedent breast cancer cells.

### PMM-172 inhibited STAT3 downstream target genes expression

STAT3 target genes are vital to the dysfunctional biology processes induced by the aberrantly activated STAT3. Therefore, we investigated whether PMM-172 suppresses the expression of STAT3-regulated genes related to cell survival, proliferation and apoptosis in MDA-MB-231 cells. As shown in Fig. 7D, the treatment with PMM-172 for 24 h down-regulated the expression of Bcl-2, Bcl-xL, survivin and cyclin D1 in a dose-dependent manner.

## Discussion

The research advance on the biological information of STAT3 has paved the way for its utilization in cancer therapy. Also, current efforts on exploiting STAT3-targeting agents have made impressive progress: an increasing number of synthetic and natural-occurring chemical entities have been reported with potent STAT3 inhibition efficiency since the last decade. Yet remarkable challenges are foreseeable transferring the testing agents into clinical drugs. Apart from the attrition in the preclinical and clinical studies, the developing of a certain chemotype into approved medicine is also likely to be plagued by resistance and relapse of tumor. In this situation, an ample multiformity in chemical space is favorable to explore for different kinds of inhibitors which shall alleviate the coming problems. Of note is that the traditional Chinese herbals provide a non-negligible candidate pool for digging of new STAT3 inhibitors. Diverse in structure, these natural products brought new understanding in the biological functional mechanism with STAT3 and novel starting points with considerable improving space^31–35^. Attempts in the characterization and optimization for these NP as STAT3 inhibitors may yield admirable outputs. Also noteworthy is that some NPs are restricted in medicinal use not only for the complexity of purification or/and synthesis, but also for the difficulty in optimization. As one of the most famous medicinal plant metabolites, SHK is unlike some other “untamed” NPs for bearing sites apt to be modified in structure. Based on the knowledge that SHK has a remarkable STAT3 inhibitory profile, it’s of interest to investigate its probable molecular mechanism and explore for optimizations.

In this study, the binding mode of SHK with STAT3 was primarily explored by virtual simulation with a high ranking score. On the basis of maintaining key intermolecular interactions with receptor STAT3, we applied the scaffold growth strategy to modify the backbone SHK and establish a small library of SHK derivatives with higher binding affinity. Iterative docking screen simulations were performed to rank all the new compounds and the top hit was validated. The hit compound PMM-158 shared a quite similar binding modality with its original scaffold SHK, while the substituent group provided additional favorable interactions. Structural modification was carried out to PMM-158 in order to gain a further understanding on the structure-activity relationship. Also, molecular dynamics was performed to the complex systems of SHK, PMM-158 and PMM-172 with protein STAT3 to detect their robustness. The fluctuant RMSD values for both systems and ligands were quite stable in the equilibrium state, hinting a high reliability of the binding models.

After the systematic synergetic simulation work, the target compounds were synthesized and tested *in vitro* to depict their pharmacological profile. According to Table 1, the obtained synthetics had lower toxicity against the non-tumorigenic MCF-10A cells than SHK. Also these compounds exerted more potent anti-proliferative activity against HER2 over-expression cell line and triple negative cell lines than the other two cell lines (shown in Table 2). Among all the entities, PMM-172 showed better anti-proliferative activity against one triple negative cell line MDA-MB-231 (IC_50_ = 1.98 ± 0.49 *μ*M) than SHK (3.28 ± 0.41 *μ*M) and Stattic (3.76 ± 0.50 *μ*M). What’s more, PMM-172 could decrease the STAT3 luciferase activity in a dose-dependent manner, and the effect was similar with Stattic.

Subsequently, we investigated the effects of PMM-172 on cell apoptosis in MDA-MB-231 cells. The results demonstrated that PMM172 induced cell apoptosis in dose- and time- dependent manners, and more effectively than Stattic. Also the elevated expression of cleaved PARP and cleaved caspase-3, which are hallmarks of cell apoptosis, were observed by the treatment with increased concentration of PMM-172. Furthermore, the depolarization of mitochondria and reduced mitochondrial transmembrane potential by PMM-172 were confirmed. Taken together, these results indicated that PMM-172 induced the cell apoptosis of MDA-MB-231 cells through the mitochondrial pathway.

For the mechanistic study, we found that PMM-172 inhibited the constitutive/inducible STAT3 activation in MDA-MB-231 cells, and showed a slight advantage over Stattic. In contrary, the level of the phosphorylated STAT3 was not affected by PMM-172 in non-cancer MCF-10A cells. The expression levels of STAT1, STAT5 and their phosphorylated forms in MDA-MB-231 cells were further detected to character the selectivity of PMM-172. While no obvious changes were observed in the phosphorylation levels of STAT1 and STAT5, a conclusion may be safely conducted that PMM-172 primarily suppressed STAT3 activation in STAT3-dependent breast cancer cells.

It has been reported that the suppression of STAT3 activation results in the reduction of nuclear localization of STAT3^36^. When exerting PMM-172 on MDA-MB-231 cells, the expression levels of STAT3 in nuclear fractions were reduced, in accordance with the mentioned reports. Also it’s known that after phosphorylation, STAT3 translocates into the nuclear where it binds to a specific promoter to regulate targeted genes expression related to cell survival, proliferation and apoptosis^37^. In our study, levels of representative downstream proteins including Bcl-2, Bcl-XL, survivin and cyclin D1 were measured to reveal that PMM-172 could down-regulate the expression of STAT3 target genes in MDA-MB-231 cells.

Collectively, these results favored our design intention and hinted this type of natural product derivatives might be helpful in the further explorations of potent STAT3 inhibitors.

## Methods

### Materials and measurements

All chemicals (reagent grade) used were purchased from Nanjing Chemical Reagent Co. Ltd. (Nanjing, China). All the ^1^H NMR spectra were recorded on a Bruker DPX 300 model Spectrometer in CDCl_3_ and chemical shifts (Δ) were reported as parts per million (ppm). ESI-MS spectra were recorded a Mariner System 5304 Mass spectrometer. Elemental analyses were performed on a CHNO-Rapid instrument and were within 0.4% of the theoretical values. Thin layer chromatography (TLC) was performed on silica gel plates (Silica Gel 60 GF254) and visualized in UV light (254 nm). Column chromatography was performed using silica gel (200–300 mesh) eluting with ethyl acetate and petroleum ether (bp. 30–60 °C).

STAT3 Antibody (#12640), phospho-STAT3 (Tyr705) Antibody (#9131) and GAPDH Antibody (#2118) were purchased from Cell Signaling Technology (Beverly, MA). BCL-XL Antibody (WL01558), Bcl-2 Antibody (WL01556), Survivin Antibody (WL01684), Cyclin D1 Antibody (WL01435a), β-actin Antibody (WL01774), Lamin B Antibody (WL01775), and Goat anti-rabbit IgG (H + L) (WLA023a) were purchased from Wanleibio Co., Ltd. (Shenyang, China). 3-(4,5-Dimethylthiazol-2-yl)-2,5-diphenyltetrazolium (MTT) were purchased from Sigma-Aldrich (St. Louis, USA). Annexin V-FITC Apoptosis Detection Kit (A211-01/02) was purchased from Vazyme Biotech Co.,Ltd (Nanjing, China).

### Docking simulation and scaffold modification

The STAT3 crystal structure (PDB code: 1BG1) was deposited from the PDB database as a dimer. Afterwards, the missing residues in STAT3 (residues 185–193, 689–701, and 717–722) were added using Modeller^38^. The target was prepared using Accelrys Discovery Studio version 3.5 as follows: (1) All the water molecules and the DNA chains were removed; (2) Hydrogens assigned to the monomer A of 1BG1 which were then applied with CHARMM force field and minimized; (3) The centroid of residue pTyr705 of the monomer B was used as the center of binding pocket. Thereafter the ligand SHK was also prepared and minimized using Discovery Studio version 3.5 and the module CDOCKER was utilized to perform the docking simulation. Based on the predicted binding mode of SHK with STAT3, the scaffold modification and following docking simulation were achieved by dint of the module Grow Scaffold in Accelrys Discovery Studio version 3.5. Following the protocol, the hydroxyl at the branching chain of SHK was marked as site to be substituted. To increase the diversity, all the reaction types provided by the software were selected. After calculation, modified molecules were produced and combined with molecules designed empirically to furnish a library of small molecules. We used CDOCKER of Discovery Studio 3.5 to dock the pre-generated conformations into STAT3 and small-molecule compounds with high scores were selected to further confirm their pharmacological activities.

### Molecular dynamics (MD) stimulations

To further verify the binding model, MD simulations were carried out using the GROMACS package (version 5.1.2). Proteins were charged using the GROMOS96 43a1p force field (which could handle with pTyr residue) and the small molecules were prepared using PRODRG. The complex was solvated in a dodecahedron water box (1-nm thick) of SPC water molecules and periodic boundary conditions were applied in all directions. Periodic boundary conditions (PBC) were employed to avoid edge effects in MD simulations. The systems were neutralized with Na^+^ and Cl^−^ counter ions and the long-range electrostatic interactions were calculated by the particle mesh Ewald method. Prior to performing the MD simulation, energy minimization was carried out to clear poor contacts. Then, 100 ps NVT and 100 ps NPT ensembles with simultaneous protein–ligand position restraints were also carried out to equilibrate the system. Consequently, 10 ns MD production simulation was performed with a 2 fs time step at constant temperature (300 K) and pressure (1 atm).

### General procedure for the synthesis of compounds PMM-158~PMM-173

Shikonin, C1-C16, 4-dimethyaminopyridine (DMAP) and *N*, *N’*-dicyclohexyl-carbodiimide (DCC) were dissolved in dichloromethane. The reaction was stirred at ice bath for 8 h and monitored by TLC. After the completion of the reaction, the product PMM-158~PMM-173 was obtained and purified by column chromatography.

### Cell Culture

Human breast cancer cells and human non-tumorigenic breast cells (MCF-10A) were purchased from Nanjing Keygen Technology (Nanjing, China). Cells were maintained in Dulbecco’s modified Eagle’s medium (DMEM, Hyclone) (High Glucose) with L-glutamine supplemented with 10% fetal bovine serum (FBS, BI), 100 U/mL penicillin and 100 mg/mL streptomycin (Hyclone), and incubated at 37 °C in a humidified atmosphere containing 5% CO_2_.

### Anti-proliferation assay

The anti-proliferative activities of the prepared compounds against MCF-7 (ER+/PR+, HER-2-), BT474 (ER+/PR+, HER-2+), SKBR-3 (ER-/PR-/HER-2+), MDA-MB-436 (ER-/PR-/HER-2-), MDA-MB-231 (ER-/PR-/HER-2-), MDA-MB-468 (ER-/PR-/HER-2-) and non-tumorigenic MCF-10A cell lines were evaluated using a standard (MTT)-based colorimetric assay with some modification. Cell lines were grown to log phase in DMEM supplemented with 10% fetal bovine serum. Cell suspensions were prepared and 100 *μ*L/well dispensed into 96-well plates giving 10^4^ cells/well. The subsequent incubation was permitted at 37 °C, 5% CO_2_ atmosphere for 24 h to allow the cells to reattach. Subsequently, cells were treated with the target compounds at increasing concentrations in the presence of 10% FBS for 24 h. Then, cell viability was assessed by the conventional 3-(4, 5-dimethylthiazol-2-yl)-2, 5-diphenyltetrazolium bromide (MTT) reduction assay and carried out strictly according to the manufacturer instructions. The absorbance (OD_570_) was read on an ELISA reader (Tecan, Austria). In all experiments, three replicate wells were used for each drug concentration. Each assay was carried out for at least three times.

### Luciferase assay

Cells were seeded in 24 well plates and transiently transfected together with STAT3 reporter plasmid 4 × M67 pTATA TK-Luc (0.2 *μ*g/per well, Addgene, USA) and Renilla luciferase control reporter plasmid pRL-CMV (0.1 *μ*g/per well, promega, USA), using lipofectamine 2000 (Invitrogen, USA). Then the cells were incubated with complete medium for 24 h with PMM-172 or Stattic. Following the treatment, the cells were harvested in 100 *μ*L of passive lysis buffer (Promega, USA) in each well. A 25 *μ*L aliquot of cell lysate was subjected to a luciferase assay by using the dual-luciferase assay kit (Promega, USA). STAT3 luciferase activity was measured by EnVision Mutilabel Reader (Perkin Elmer, USA). Relative luciferase activity was calculated after the activity of STAT3-dependent firefly luciferase had been normalized to that of Renilla luciferase. All values are expressed as - fold induction relative to basal activity.

### Cell apoptosis assay

Approximately 10^5^ cells/well were plated in a 24 well plate and allowed to adhere. Subsequently, the medium was replaced with fresh culture medium containing PMM-172 or Stattic. Non-treated wells received an equivalent volume of ethanol (<0.1%). After 24 h, they were trypsinized, washed in PBS and centrifuged at 2000 rpm for 5 min. The pellet was then resuspended in 500 *μ*L of staining solution (containing 5 *μ*L AnnexinV-FITC and 5 *μ*L PI in Binding Buffer), mixed gently and incubated for 15 min at room temperature in dark. The samples were then analyzed by a FACSCalibur flow cytometer (Becton Dickinson, San Jose, CA, USA).

### Mitochondrial membrane potential evaluation

Mitochondrial transmembrane potential (∆*Ψ*m) was detected using a JC-1 mitochondrial membrane potential assay kit (Beyotime Biotech), following the manufacturer’s protocol. After the treatment, the cells were incubated at 37 °C for 20 min with 5 *μ*g/mL JC-1 (5,5,6,6-tetrachloro-1,1,3,3- tetraethylbenzimidazolylcarbocyanine iodide), then washed twice with PBS and placed in fresh medium without serum. The samples were analyzed using a FACSCalibur cytometer (Becton Dickinson) equipped with a 488 nm argon laser.

### Preparation of cytoplasmic and nuclear extracts

To examine the relative proportion of STAT3 in the cytoplasm and nucleus, cytoplasmic and nuclear extracts were prepared. In brief, control and treated cells were collected and suspended in a buffer containing 10 mM HEPES (pH 7.9), 10 mM KCl, 0.1 mM EDTA (pH 8.0), 0.1 mM EGTA (pH 7.0), 1 mM DTT, 0.5 mM PMSF, 2 *μ*g/mL leupeptin, 2 *μ*g/mL aprotonin, 0.5 mg/mL benzamidine. After 15 min, NP-40 (0.25%) was added, and cell lysate was vortexed for 10 s and centrifuged at 10000 rmp at 4 °C for 1 min. The supernatants were transferred to fresh tubes and considered as cytoplasmic extract. The pellets were resuspended in a buffer containing 20 mM HEPES (pH 7.9), 400 mM NaCl, 1 mM EDTA (pH 8.0), 1 mM EGTA (pH 7.0), 1 mM DTT, 1 mM PMSF, 2 *μ*g/mL leupeptin, 2 *μ*g/mL aprotonin, 0.5 mg/mL benzamidine. After 30 min incubation on ice, the extract was centrifuged at 14000 rmp at 4 °C for 10 min, and the resultant supernatants were collected as nuclear extracts. The protein content in the nuclear and cytoplasmic fractions was determined using a Pierce BCA protein assay kit.

### Western blot analysis

Cells were rinsed twice with cold PBS and then lysed with RIPA buffer containing a protease inhibitor mixture at 1:100 dilution on ice for 30 min. Insoluble components of cell lysates were removed by centrifugation (4 °C, 12000 rmp, 10 min), and protein concentrations were measured using a Pierce BCA protein assay kit. Proteins were separated by sodium dodecyl sulfate-polyacrylamide gel electrophoresis (SDS-PAGE) and transferred from the gel onto polyvinylidene difluoride (PVDF) membranes. The membranes were blocked with 5% skim milk in TBST buffer for 1 h at room temperature, and then incubated with corresponding primary antibodies diluted in 5% milk-TBST solution at 4 °C with gentle shaking overnight. After washing five times (5 min each), corresponding HRP-conjugated secondary antibodies were incubated for 1 h at room temperature. Immunoreactive bands were visualized using an ECL detection kit (Invitrogen, USA) following the manufacturer’s instruction.

### IL-6 induction of STAT3 phosphorylation

Cells were seeded in 10 cm plates and allowed to adhere overnight. The following day, the specimen was serum starved for 24 h. The specimen was then treated with or without tested agents at the dose of 2 *μ*M. After 24 h, the treated specimen was stimulated with IL-6 (25 ng/mL). Cells were then harvested after 30 min and analyzed by Western blot.

### Statistical analysis

All data are expressed as mean ± SD. The statistical analysis was performed by the Student’s t-test, using the statistical software OriginPro 2015. P values < 0.05 were considered statistically significant.

## Electronic supplementary material

Identification of New Shikonin Derivatives as Antitumor Agents Targeting STAT3 SH2 Domain
