# Supplementary material for: Identification of New Shikonin Derivatives as Antitumor Agents Targeting STAT3 SH2 Domain
Source: Sci Rep. 2017 Jun 6;7:2863. doi: 10.1038/s41598-017-02671-7 (PMC5460289; doi:10.1038/s41598-017-02671-7)
Supplement: Supplementary file 1 — Identification of New Shikonin Derivatives as Antitumor Agents Targeting STAT3 SH2 Domain [file 41598_2017_2671_MOESM1_ESM.doc]

**Identification of New Shikonin Derivatives as Antitumor Agents Targeting STAT3 SH2 Domain**

**Han-Yue Qiu ^a,b†^, Xiang Zhu ^a,b†^, Yue-Lin Luo ^a,b^, Hong-Yan Lin ^a,b^, Cheng-Yi Tang ^a,b^, Jin-Liang Qi ^a,b^, Yan-Jun Pang ^a^, Rong-Wu Yang ^a^, Gui-Hua Lu ^a,b*^, Xiao-Ming Wang ^a,b^*, Yong-Hua Yang ^a,b^***

*^a^State Key Laboratory of Pharmaceutical Biotechnology, NJU-NJFU Joint Institute of Plant Molecular Biology, Nanjing University, Nanjing 210023, China*

*^b^Co-Innovation Center for Sustainable Forestry in Southern China, Nanjing Forestry University, Nanjing 210037, China*

* Corresponding authors:

Tel./fax: +86-25-89686305

E-mail address: yangyh@nju.edu.cn, wangxm07@nju.edu.cn, guihua.lu@nju.edu.cn

**^†^**These two authors contributed equally to this paper.

*(R)-1-(5,8-dihydroxy-1,4-dioxo-1,4-dihydronaphthalen-2-yl)-4-methylpent-3-en-1-yl 2-((5-phenyl-1,3,4-oxadiazol-2-yl)thio)acetate (* ***PMM-158*** *)*

Red oil, yield 56%; ^1^H NMR (300 MHz, CDCl_3_) *δ* 12.58 (s, 1H, -OH), 12.40 (s, 1H, -OH), 8.00 (dd, *J* = 7.9, 1.7 Hz, 2H, Ar-H), 7.58 – 7.46 (m, 3H, Ar-H), 7.18 (s, 2H, Ar-H), 7.10 (d, *J* = 0.9 Hz, 1H, Ar-H), 6.14 (dd, *J* = 6.5, 5.2 Hz, 1H, -CH=C), 5.11 (t, *J* = 7.3 Hz, 1H, -CH), 4.20 (s, 2H, -CH_2_), 2.61 (ddd, *J* = 22.2, 14.8, 7.9 Hz, 2H, -CH_2_), 1.68 (s, 3H, -CH_3_), 1.58 (s, 3H, -CH_3_). MS (ESI): 507.1 ([M+H]^+^). Anal. Calcd for C_26_H_22_N_2_O_7_S: C, 61.65; H, 4.38; N, 5.53; Found: C, 61.63; H, 4.37; N, 5.51.

*(R)-1-(5,8-dihydroxy-1,4-dioxo-1,4-dihydronaphthalen-2-yl)-4-methylpent-3-en-1-yl 2-((5-(4-fluorophenyl)-1,3,4-oxadiazol-2-yl)thio)acetate (* ***PMM-159*** *)*

Red oil, yield 51%; ^1^H NMR (300 MHz, CDCl_3_) *δ* 12.55 (s, 1H, -OH), 12.37 (d, *J* = 2.3 Hz, 1H, -OH), 7.98 (ddd, *J* = 9.7, 5.1, 2.5 Hz, 2H, Ar-H), 7.20 (td, *J* = 4.3, 1.6 Hz, 1H, Ar-H), 7.17 (q, *J* = 1.6 Hz, 1H, Ar-H), 7.15 (d, *J* = 3.2 Hz, 2H, Ar-H), 7.07 (d, *J* = 0.9 Hz, 1H, Ar-H), 6.16 – 6.05 (m, 1H,-CH=C), 5.16 – 5.03 (m, 1H, -CH), 4.17 (s, 2H, -CH_2_), 2.74 – 2.50 (m, 2H, -CH_2_), 1.66 (s, 3H, -CH_3_), 1.56 (s, 3H, -CH_3_). MS (ESI): 525.1 ([M+H]^+^). Anal. Calcd for C_26_H_21_FN_2_O_7_S: C, 59.54; H, 4.04; N, 5.34; Found: C, 59.53; H, 4.05; N, 5.36.

*(R)-1-(5,8-dihydroxy-1,4-dioxo-1,4-dihydronaphthalen-2-yl)-4-methylpent-3-en-1-yl 2-((5-(3-fluorophenyl)-1,3,4-oxadiazol-2-yl)thio)acetate (* ***PMM-160*** *)*

Red oil, yield 47%; ^1^H NMR (300 MHz, CDCl_3_) *δ* 12.55 (s, 1H, -OH), 12.37 (d, *J* = 2.3 Hz, 1H, -OH), 7.80 – 7.74 (m, 1H, Ar-H), 7.71 – 7.64 (m, 1H, Ar-H), 7.52 – 7.42 (m, 1H, Ar-H), 7.24 – 7.18 (m, 1H, Ar-H), 7.18 – 7.14 (m, 2H, Ar-H), 7.07 (d, *J* = 0.9 Hz, 1H, Ar-H), 6.17 – 6.07 (m, 1H, -CH=C), 5.14 – 5.03 (m, 1H, -CH), 4.18 (s, 2H, -CH_2_), 2.72 – 2.51 (m, 2H, -CH_2_), 1.66 (d, *J* = 4.0 Hz, 3H, -CH_3_), 1.57 (s, 3H, -CH_3_). MS (ESI): 525.1 ([M+H]^+^). Anal. Calcd for C_26_H_21_FN_2_O_7_S: C, 59.54; H, 4.04; N, 5.34; Found: C, 59.55; H, 4.06; N, 5.35.

*(R)-1-(5,8-dihydroxy-1,4-dioxo-1,4-dihydronaphthalen-2-yl)-4-methylpent-3-en-1-yl 2-((5-(2-fluorophenyl)-1,3,4-oxadiazol-2-yl)thio)acetate (* ***PMM-161*** *)*

Red oil, yield 61%; ^1^H NMR (300 MHz, CDCl_3_) *δ* 12.56 (s, 1H, -OH), 12.39 (s, 1H, -OH), 8.00 (ddd, *J* = 9.0, 6.4, 2.6 Hz, 1H, Ar-H), 7.53 (tdd, *J* = 8.4, 5.0, 1.8 Hz, 1H, Ar-H), 7.32 – 7.27 (m, 1H, Ar-H), 7.24 – 7.18 (m, 1H, Ar-H), 7.16 (s, 2H, Ar-H), 7.07 (d, *J* = 0.8 Hz, 1H, Ar-H), 6.12 (dd, *J* = 6.4, 5.0 Hz, 1H, -CH=C), 5.09 (t, *J* = 7.3 Hz, 1H, -CH), 4.18 (s, 2H, -CH_2_), 2.71 – 2.50 (m, 2H, -CH_2_), 1.67 (s, 3H, -CH_3_), 1.57 (s, 3H, -CH_3_). MS (ESI): 525.1 ([M+H]^+^). Anal. Calcd for C_26_H_21_FN_2_O_7_S: C, 59.54; H, 4.04; N, 5.34; Found: C, 59.56; H, 4.05; N, 5.36.

*(R)-1-(5,8-dihydroxy-1,4-dioxo-1,4-dihydronaphthalen-2-yl)-4-methylpent-3-en-1-yl 2-((5-(4-chlorophenyl)-1,3,4-oxadiazol-2-yl)thio)acetate (* ***PMM-162*** *)*

Red oil, yield 62%; ^1^H NMR (300 MHz, CDCl_3_) *δ* 12.58 (d, *J* = 15.1 Hz, 1H, -OH), 12.38 (s, 1H, -OH), 7.91 (d, *J* = 8.6 Hz, 2H, Ar-H), 7.51 – 7.40 (m, 2H, Ar-H), 7.16 (s, 2H, Ar-H), 7.05 (d, *J* = 9.9 Hz, 1H, Ar-H), 6.11 (dd, *J* = 6.5, 5.1 Hz, 1H, -CH=C), 5.09 (t, *J* = 7.2 Hz, 1H, -CH), 4.17 (s, 2H, -CH_2_), 2.71 – 2.50 (m, 2H, -CH_2_), 1.67 (s, 3H, -CH_3_), 1.58 (d, *J* = 8.3 Hz, 3H, -CH_3_). MS (ESI): 542.1 ([M+H]^+^). Anal. Calcd for C_26_H_21_ClN_2_O_7_S: C, 57.73; H, 3.91; N, 5.18; Found: C, 57.74; H, 3.92; N, 5.16.

*(R)-1-(5,8-dihydroxy-1,4-dioxo-1,4-dihydronaphthalen-2-yl)-4-methylpent-3-en-1-yl 2-((5-(3-chlorophenyl)-1,3,4-oxadiazol-2-yl)thio)acetate (* ***PMM-163*** *)*

Red oil, yield 44%; ^1^H NMR (300 MHz, CDCl_3_) *δ* 12.55 (s, 1H, -OH), 12.37 (s, 1H, -OH), 7.97 (dt, *J* = 7.3, 1.6 Hz, 1H, Ar-H), 7.90 – 7.83 (m, 1H, Ar-H), 7.53 – 7.36 (m, 2H, Ar-H), 7.15 (s, 2H, Ar-H), 7.06 (d, *J* = 0.8 Hz, 1H, Ar-H), 6.16 – 6.06 (m, 1H, -CH=C), 5.14 – 5.03 (m, 1H, -CH), 4.18 (s, 2H, -CH_2_), 2.71 – 2.45 (m, 2H, -CH_2_), 1.65 (d, *J* = 8.7 Hz, 3H, -CH_3_), 1.57 (s, 3H, -CH_3_). MS (ESI): 542.1 ([M+H]^+^). Anal. Calcd for C_26_H_21_ClN_2_O_7_S: C, 57.73; H, 3.91; N, 5.18; Found: C, 57.75; H, 3.92; N, 5.17.

*(R)-1-(5,8-dihydroxy-1,4-dioxo-1,4-dihydronaphthalen-2-yl)-4-methylpent-3-en-1-yl 2-((5-(2-chlorophenyl)-1,3,4-oxadiazol-2-yl)thio)acetate (* ***PMM-164*** *)*

Red oil, yield 63%; ^1^H NMR (300 MHz, CDCl_3_) *δ* 12.56 (s, 1H, -OH), 12.39 (s, 1H, -OH), 7.94 (ddd, *J* = 7.8, 3.9, 1.9 Hz, 1H, Ar-H), 7.57 – 7.34 (m, 4H, Ar-H), 7.16 (s, 2H, Ar-H), 6.12 (dd, *J* = 6.6, 5.3 Hz, 1H, -CH=C), 5.10 (t, *J* = 7.2 Hz, 1H, -CH), 4.19 (s, 2H, -CH_2_), 2.73 – 2.54 (m, 2H, -CH_2_), 1.67 (s, 3H, -CH_3_), 1.57 (s, 3H, -CH_3_). MS (ESI): 542.1 ([M+H]^+^). Anal. Calcd for C_26_H_21_ClN_2_O_7_S: C, 57.73; H, 3.91; N, 5.18; Found: C, 57.74; H, 3.93; N, 5.16.

*(R)-1-(5,8-dihydroxy-1,4-dioxo-1,4-dihydronaphthalen-2-yl)-4-methylpent-3-en-1-yl 2-((5-(4-bromophenyl)-1,3,4-oxadiazol-2-yl)thio)acetate (* ***PMM-165*** *)*

Red oil, yield 56%; ^1^H NMR (300 MHz, CDCl_3_) *δ* 12.55 (d, *J* = 2.3 Hz, 1H, -OH), 12.37 (d, *J* = 2.4 Hz, 1H, -OH), 7.89 – 7.79 (m, 2H, Ar-H), 7.67 – 7.58 (m, 2H, Ar-H), 7.16 (s, 2H, Ar-H), 7.06 (d, *J* = 0.9 Hz, 1H, Ar-H), 6.18 – 6.05 (m, 1H, -CH=C), 5.09 (t, *J* = 7.3 Hz, 1H, -CH), 4.15 (d, *J* = 12.6 Hz, 2H, -CH_2_), 2.74 – 2.41 (m, 2H, -CH_2_), 1.64 (d, *J* = 14.2 Hz, 3H, -CH_3_), 1.57 (s, 3H, -CH_3_). MS (ESI): 586.0 ([M+H]^+^). Anal. Calcd for C_26_H_21_BrN_2_O_7_S: C, 53.34; H, 3.62; N, 4.79; Found: C, 53.35; H, 3.63; N, 4.77.

*(R)-1-(5,8-dihydroxy-1,4-dioxo-1,4-dihydronaphthalen-2-yl)-4-methylpent-3-en-1-yl 2-((5-(3-bromophenyl)-1,3,4-oxadiazol-2-yl)thio)acetate (* ***PMM-166*** *)*

Red oil, yield 62%; ^1^H NMR (300 MHz, CDCl_3_) *δ* 12.54 (d, *J* = 7.7 Hz, 1H, -OH), 12.38 (s, 1H, -OH), 7.92 (d, *J* = 7.9 Hz, 2H, Ar-H), 7.16 (s, 3H, Ar-H), 7.07 (s, 1H, Ar-H), 6.91 (s, 1H, Ar-H), 6.12 (s, 1H, -CH=C), 5.14 – 5.07 (m, 1H, -CH), 4.18 (s, 2H, -CH_2_), 2.36 (t, *J* = 7.5 Hz, 2H, -CH_2_), 1.67 (s, 3H, -CH_3_), 1.57 (s, 3H, -CH_3_). MS (ESI): 586.0 ([M+H]^+^). Anal. Calcd for C_26_H_21_BrN_2_O_7_S: C, 53.34; H, 3.62; N, 4.79; Found: C, 53.36; H, 3.64; N, 4.78.

*(R)-1-(5,8-dihydroxy-1,4-dioxo-1,4-dihydronaphthalen-2-yl)-4-methylpent-3-en-1-yl 2-((5-(2-bromophenyl)-1,3,4-oxadiazol-2-yl)thio)acetate (* ***PMM-167*** *)*

Red oil, yield 68%; ^1^H NMR (300 MHz, CDCl_3_) *δ* 12.56 (s, 1H, -OH), 12.39 (d, *J* = 2.3 Hz, 1H, -OH), 7.88 (dd, *J* = 7.7, 1.8 Hz, 1H, Ar-H), 7.73 (dd, *J* = 7.9, 1.2 Hz, 1H, Ar-H), 7.48 – 7.36 (m, 2H, Ar-H), 7.17 (s, 2H, Ar-H), 7.08 (d, *J* = 0.8 Hz, 1H, Ar-H), 6.13 (dd, *J* = 6.5, 5.3 Hz, 1H, -CH=C), 5.10 (t, *J* = 7.3 Hz, 1H, -CH), 4.19 (d, *J* = 0.6 Hz, 2H, -CH_2_), 2.60 (ddd, *J* = 22.7, 15.0, 8.3 Hz, 2H, -CH_2_), 1.68 (s, 3H, -CH_3_), 1.57 (s, 3H, -CH_3_). MS (ESI): 586.0 ([M+H]^+^). Anal. Calcd for C_26_H_21_BrN_2_O_7_S: C, 53.34; H, 3.62; N, 4.79; Found: C, 53.35; H, 3.63; N, 4.80.

*(R)-1-(5-hydroxy-1,4-dioxo-1,4-dihydronaphthalen-2-yl)-4-methylpent-3-en-1-yl 2-((5-(4-methoxyphenyl)-1,3,4-oxadiazol-2-yl)thio)acetate (* ***PMM-168*** *)*

Red oil, yield 63%; ^1^H NMR (300 MHz, CDCl_3_) *δ* 12.55 (d, *J* = 3.3 Hz, 1H, -OH), 12.39 (s, 1H, -OH), 7.92 (dd, *J* = 11.6, 6.1 Hz, 6H, Ar-H), 7.06 (d, *J* = 9.0 Hz, 1H, Ar-H), 6.16 – 6.06 (m, 1H, -CH=C), 5.09 (s, 1H, -CH), 4.26 (q, *J* = 7.1 Hz, 2H, -CH_2_), 2.71 – 2.45 (m, 3H, -OCH_3_), 2.40 – 2.26 (m, 2H, -CH_2_), 1.66 (s, 3H, -CH_3_), 1.56 (s, 3H, -CH_3_). MS (ESI): 521.1 ([M+H]^+^). Anal. Calcd for C_27_H_24_N_2_O_7_S: C, 62.30; H, 4.65; N, 5.38; Found: C, 62.32; H, 4.66; N, 5.37.

*(R)-1-(5,8-dihydroxy-1,4-dioxo-1,4-dihydronaphthalen-2-yl)-4-methylpent-3-en-1-yl 2-((5-(3-methoxyphenyl)-1,3,4-oxadiazol-2-yl)thio)acetate (* ***PMM-169*** *)*

Red oil, yield 56%; ^1^H NMR (300 MHz, CDCl_3_) *δ* 12.56 (s, 1H, -OH), 12.38 (s, 1H, -OH), 7.56 (d, *J* = 1.0 Hz, 1H, Ar-H), 7.53 (s, 1H, Ar-H), 7.51 – 7.48 (m, 1H, Ar-H), 7.44 – 7.39 (m, 1H, Ar-H), 7.37 (d, *J* = 7.8 Hz, 1H, Ar-H), 7.16 (s, 2H, Ar-H), 6.16 – 6.06 (m, 1H, -CH=C), 5.09 (t, *J* = 7.3 Hz, 1H, -CH), 4.17 (s, 2H, -CH_2_), 3.87 (d, *J* = 2.7 Hz, 3H, -OCH_3_), 2.74 – 2.50 (m, 2H, -CH_2_), 1.67 (s, 3H, -CH_3_), 1.57 (s, 3H, -CH_3_). MS (ESI): 521.1 ([M+H]^+^). Anal. Calcd for C_27_H_24_N_2_O_7_S: C, 62.30; H, 4.65; N, 5.38; Found: C, 62.29; H, 4.64; N, 5.36.

*(R)-1-(5,8-dihydroxy-1,4-dioxo-1,4-dihydronaphthalen-2-yl)-4-methylpent-3-en-1-yl 2-((5-(2-methoxyphenyl)-1,3,4-oxadiazol-2-yl)thio)acetate (* ***PMM-170*** *)*

Red oil, yield 61%; ^1^H NMR (300 MHz, CDCl_3_) *δ* 12.55 (s, 1H, -OH), 12.38 (s, 1H, -OH), 7.85 (dd, *J* = 7.6, 1.7 Hz, 1H, Ar-H), 7.48 (ddd, *J* = 9.1, 6.4, 1.7 Hz, 1H, Ar-H), 7.16 (s, 2H, Ar-H), 7.06 (s, 1H, Ar-H), 7.03 (d, *J* = 0.8 Hz, 1H, Ar-H), 7.01 (d, *J* = 1.8 Hz, 1H, Ar-H), 6.12 (dd, *J* = 6.2, 5.0 Hz, 1H, -CH=C), 5.09 (t, *J* = 7.3 Hz, 1H, -CH), 4.21 – 4.12 (m, 2H, -CH_2_), 3.94 (d, *J* = 4.7 Hz, 3H, -OCH_3_), 2.61 (ddd, *J* = 25.1, 14.9, 6.7 Hz, 2H, -CH_2_), 1.66 (s, 3H, -CH_3_), 1.56 (s, 3H, -CH_3_). MS (ESI): 521.1 ([M+H]^+^). Anal. Calcd for C_27_H_24_N_2_O_7_S: C, 62.30; H, 4.65; N, 5.38; Found: C, 62.31; H, 4.66; N, 5.37.

*(R)-1-(5,8-dihydroxy-1,4-dioxo-1,4-dihydronaphthalen-2-yl)-4-methylpent-3-en-1-yl 2-((5-(3,4-dimethoxyphenyl)-1,3,4-oxadiazol-2-yl)thio)acetate (* ***PMM-171*** *)*

Red oil, yield 57%; ^1^H NMR (300 MHz, CDCl_3_) *δ* 12.54 (s, 1H, -OH), 12.37 (s, 1H, -OH), 7.54 – 7.49 (m, 1H, Ar-H), 7.47 (d, *J* = 1.9 Hz, 1H, Ar-H), 7.14 (s, 2H, Ar-H), 7.05 (d, *J* = 0.6 Hz, 1H, Ar-H), 6.91 (d, *J* = 8.4 Hz, 1H, Ar-H), 6.12 (dd, *J* = 6.4, 5.2 Hz, 1H, -CH=C), 5.09 (t, *J* = 7.3 Hz, 1H, -CH), 4.15 (s, 2H, -CH_2_), 3.95 (t, *J* = 2.8 Hz, 6H, -OCH_3_), 2.72 – 2.42 (m, 2H, -CH_2_), 1.66 (s, 3H, -CH_3_), 1.56 (s, 3H, -CH_3_). MS (ESI): 567.1 ([M+H]^+^). Anal. Calcd for C_28_H_26_N_2_O_7_S: C, 59.36; H, 4.63; N, 4.94; Found: C, 59.34; H, 4.65; N, 4.95.

*(R)-1-(5,8-dihydroxy-1,4-dioxo-1,4-dihydronaphthalen-2-yl)-4-methylpent-3-en-1-yl 2-((5-(3,5-dimethoxyphenyl)-1,3,4-oxadiazol-2-yl)thio)acetate (* ***PMM-172*** *)*

Red oil, yield 53%; ^1^H NMR (300 MHz, CDCl_3_) *δ* 12.55 (s, 1H, -OH), 12.36 (s, 1H, -OH), 7.17 – 7.11 (m, 3H, Ar-H), 7.09 (d, *J* = 2.3 Hz, 2H, Ar-H), 7.06 (d, *J* = 0.9 Hz, 1H, Ar-H), 6.17 – 6.06 (m, 1H, -CH=C), 5.15 – 5.02 (m, 1H, -CH), 4.16 (s, 2H, -CH_2_), 3.84 (s, 6H, -OCH_3_), 2.72 – 2.50 (m, 2H, -CH_2_), 1.67 (s, 3H, -CH_3_), 1.56 (s, 3H, -CH_3_). MS (ESI): 567.1 ([M+H]^+^). Anal. Calcd for C_28_H_26_N_2_O_7_S: C, 59.36; H, 4.63; N, 4.94; Found: C, 59.35; H, 4.65; N, 4.96.

*(R)-1-(5,8-dihydroxy-1,4-dioxo-1,4-dihydronaphthalen-2-yl)-4-methylpent-3-en-1-yl 2-((5-(p-tolyl)-1,3,4-oxadiazol-2-yl)thio)acetate (* ***PMM-173*** *)*

Red oil, yield 62%; ^1^H NMR (300 MHz, CDCl_3_) δ 12.55 (s, 1H, -OH), 12.38 (s, 1H, -OH), 7.87 (t, *J* = 8.2 Hz, 4H, Ar-H), 7.31 (s, 1H, Ar-H), 7.29 (s, 1H, Ar-H), 7.16 (s, 1H, Ar-H), 5.98 (d, *J* = 1.6 Hz, 1H, -CH=C), 5.30 (s, 1H, -CH), 3.81 (s, 2H, -CH_2_), 2.42 (d, *J* = 2.6 Hz, 3H, -CH_3_), 2.35 (t, *J* = 7.5 Hz, 2H, -CH_2_), 1.65 (d, *J* = 6.1 Hz, 3H, -CH_3_), 1.56 (s, 3H, -CH_3_). MS (ESI): 521.1 ([M+H]^+^). Anal. Calcd for C_27_H_24_N_2_O_7_S: C, 62.30; H, 4.65; N, 5.38; Found: C, 62.32; H, 4.64; N, 5.40.

Supplementary Figure 1：Full-length blots of Figure 4 (E) in the main text.


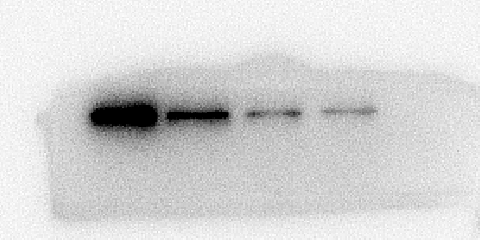

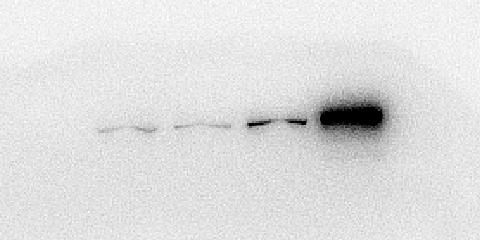


PARP Cleaved-PARP


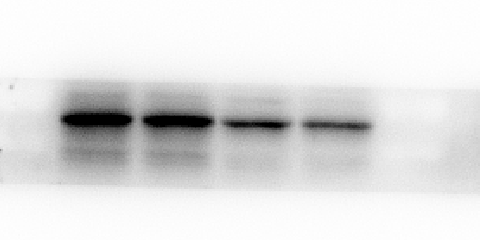

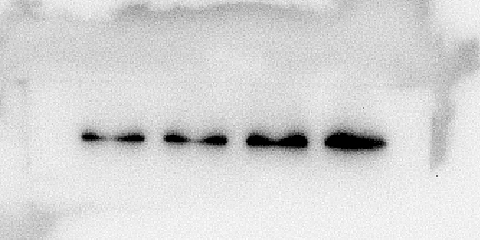


Caspase 3 Cleaved -Caspase 3


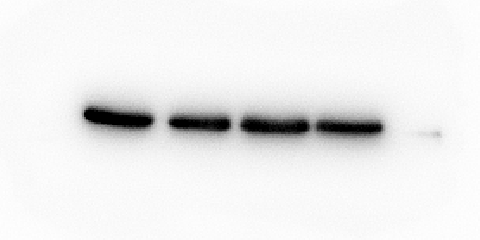


GAPDH

Supplementary Figure 2：Full-length blots of Figure 6 in the main text.

Figure 6 (A)


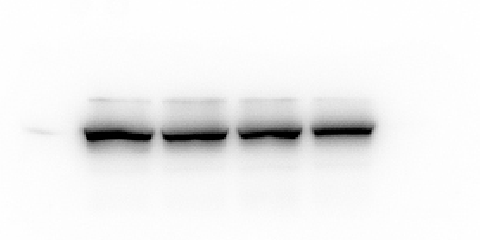

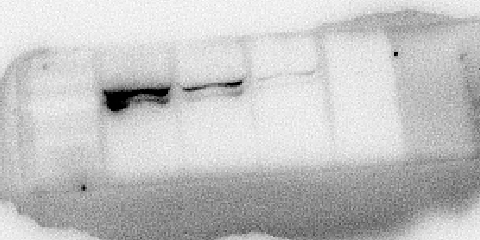


STAT3 pSTAT3 (Y705)


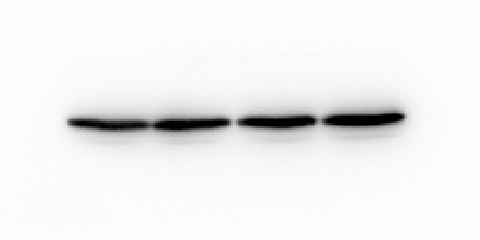


GAPDH

Figure 6 (B)


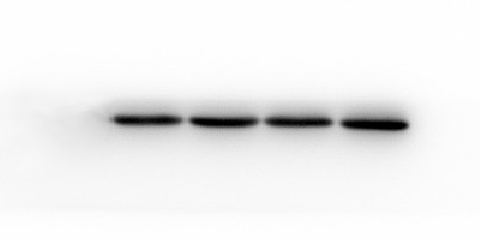

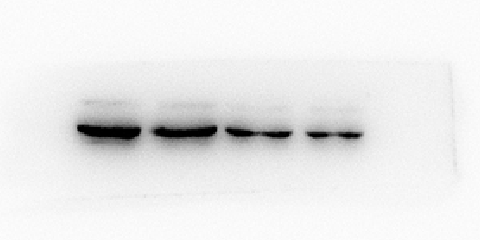


STAT3 pSTAT3 (Y705)


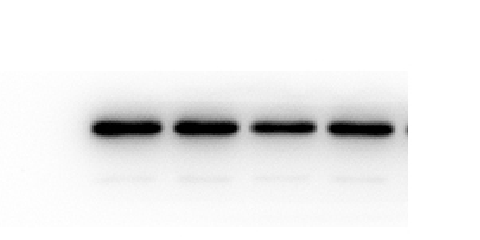


GAPDH

Figure 6 (C)


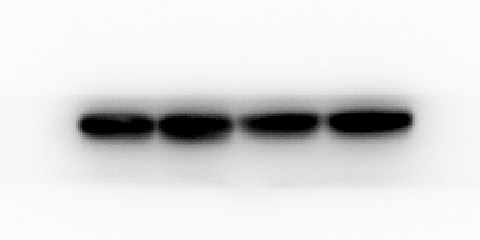

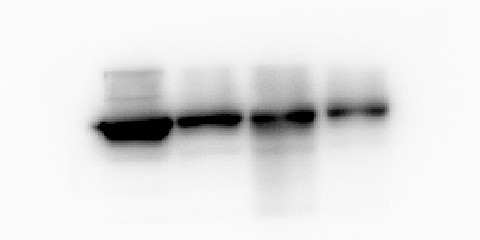


STAT3 pSTAT3 (Y705)


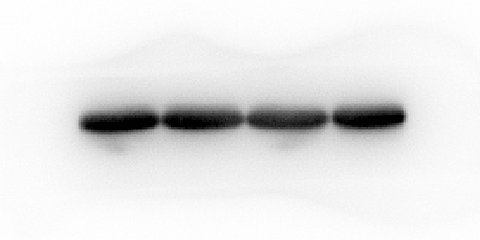


GAPDH

Figure 6 (D)


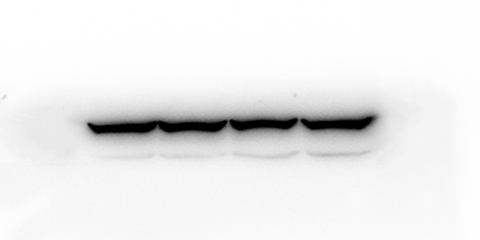

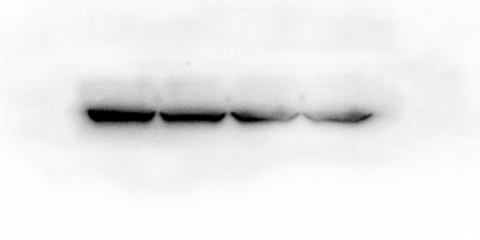


STAT3 pSTAT3 (Y705)


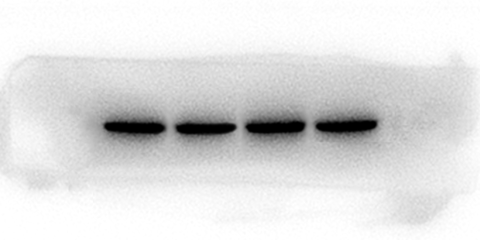


GAPDH

Figure 6 (E)


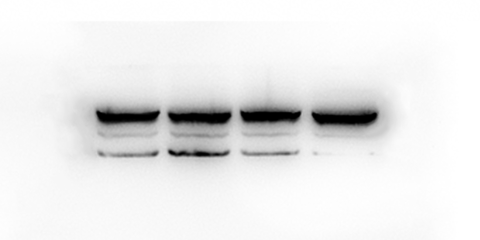

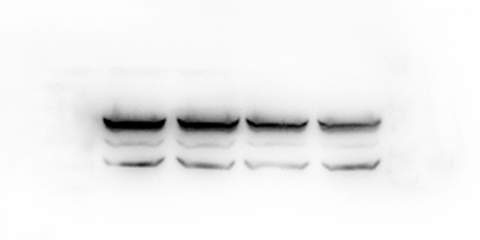


STAT3 pSTAT3 (Y705)


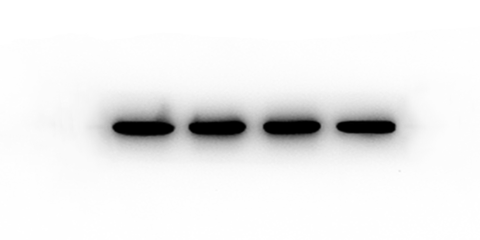


GAPDH

Figure 6 (F)


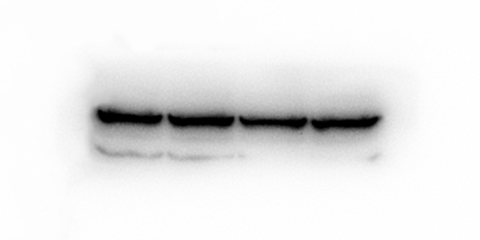

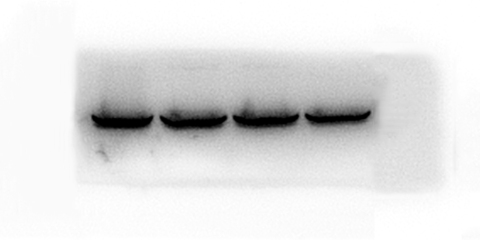


STAT1 pSTAT1


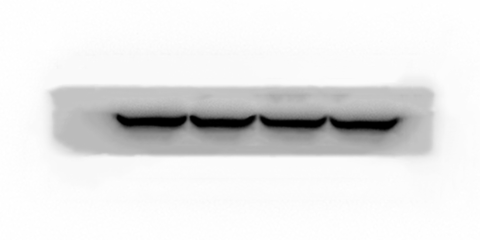

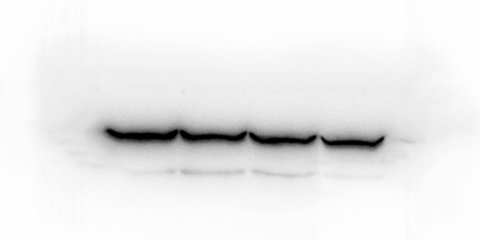


STAT5 pSTAT5


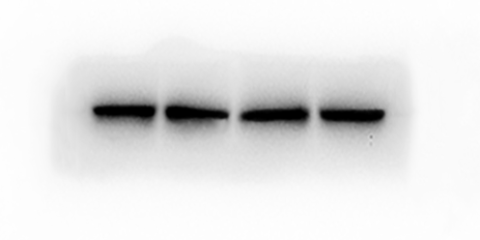


GAPDH

Supplementary Figure 3：Full-length blots of Figure 7 in the main text.

Figure 7 (A)


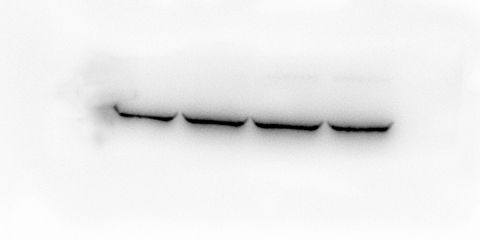

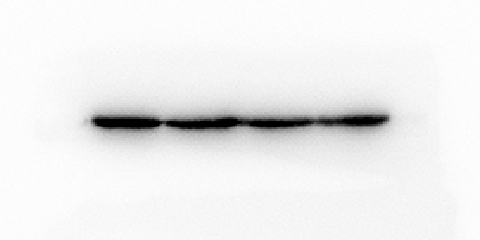


STAT3 (cytoplasmic) β-actin


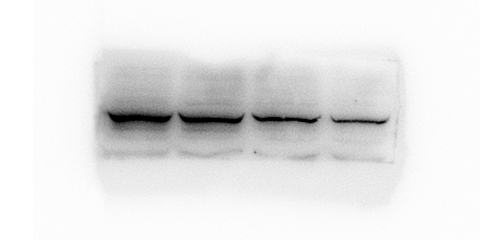

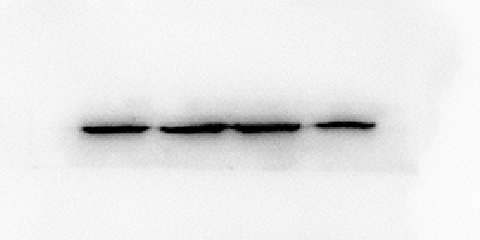


STAT3 (nuclear) Lamin B1

Figure 7 (B)


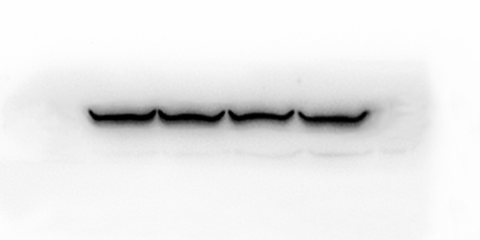

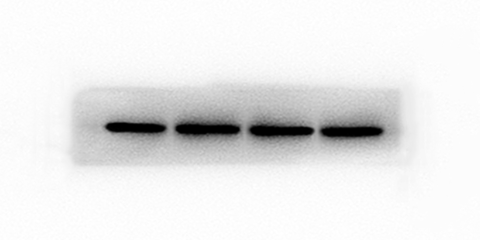


STAT3 (cytoplasmic) β-actin


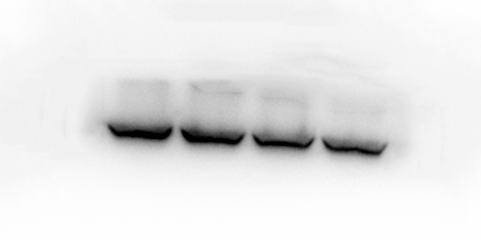

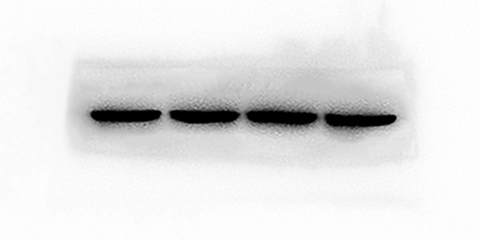


STAT3 (nuclear) Lamin B1

Figure 7 (C)


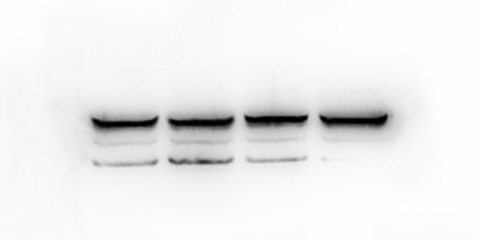

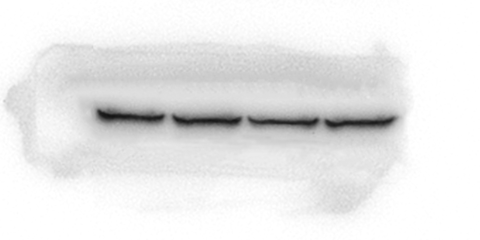


STAT3 pSTAT3 (Y705)


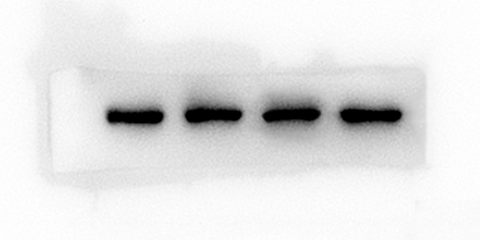


GAPDH

Figure 7 (D)


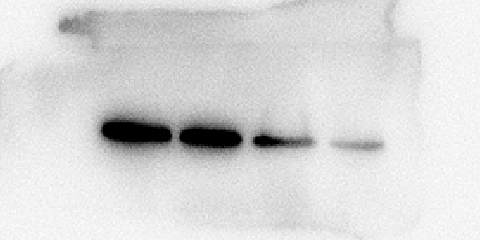

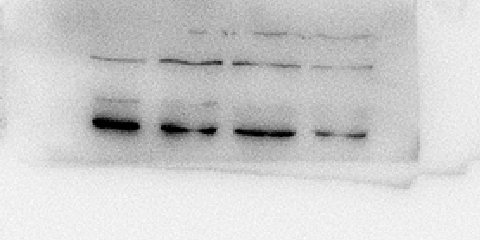


Bcl-2 Bcl-XL


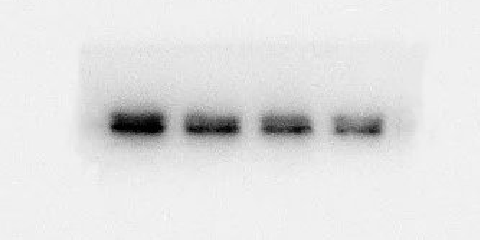

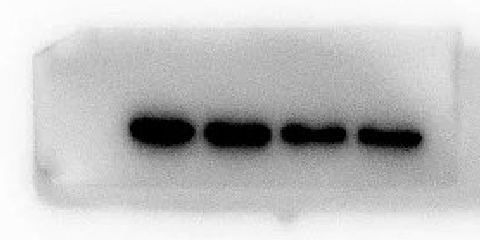


Survivin Cyclin D1


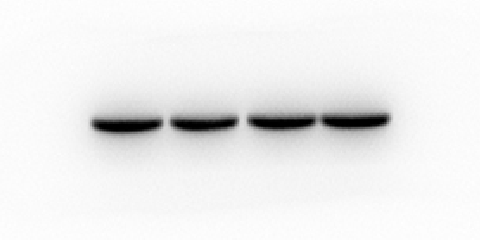


GAPDH
